# Maize DNA Methylation in Response to Drought Stress Is Involved in Target Gene Expression and Alternative Splicing

**DOI:** 10.3390/ijms22158285

**Published:** 2021-07-31

**Authors:** Qi Wang, Jie Xu, Xuemei Pu, Haozhe Lv, Yanjun Liu, Huili Ma, Fengkai Wu, Qingjun Wang, Xuanjun Feng, Tianhong Liu, Qi Tang, Yaxi Liu, Yanli Lu

**Affiliations:** 1 State Key Laboratory of Crop Gene Exploration and Utilization in Southwest China, Sichuan Agricultural University, Chengdu 611130, China; qiwang1993@gmail.com (Q.W.); jiexu28@gmail.com (J.X.); xuemeipu@163.com (X.P.); lhzsicau@126.com (H.L.); yanjunl0319@163.com (Y.L.); huilima2021@163.com (H.M.); wfk0909@163.com (F.W.); wdqdjm@126.com (Q.W.); xuanjun_feng@163.com (X.F.); jinqiangwei100@163.com (T.L.); qitang927@gmail.com (Q.T.); liuyaxi@sicau.edu.cn (Y.L.); 2 Maize Research Institute, Sichuan Agricultural University, Chengdu 611130, China; 3 Key Laboratory of Biology and Genetic Improvement of Maize in Southwest Region, Ministry of Agriculture, Chengdu 611130, China; 4 Triticeae Research Institute and Key Lab for Major Crop Diseases, Sichuan Agricultural University, Chengdu 611130, China

**Keywords:** DNA methylation, drought stress, gene regulation, maize

## Abstract

DNA methylation is important for plant growth, development, and stress response. To understand DNA methylation dynamics in maize roots under water stress (WS), we reanalyzed DNA methylation sequencing data to profile DNA methylation and the gene expression landscape of two inbred lines with different drought sensitivities, as well as two of their derived recombination inbred lines (RILs). Combined with genotyping-by-sequencing, we found that the inheritance pattern of DNA methylation between RILs and parental lines was sequence-dependent. Increased DNA methylation levels were observed under WS and the methylome of drought-tolerant inbred lines were much more stable than that of the drought-sensitive inbred lines. Distinctive differentially methylated genes were found among diverse genetic backgrounds, suggesting that inbred lines with different drought sensitivities may have responded to stress in varying ways. Gene body DNA methylation showed a negative correlation with gene expression but a positive correlation with exon splicing events. Furthermore, a positive correlation of a varying extent was observed between small interfering RNA (siRNA) and DNA methylation, which at different genic regions. The response of siRNAs under WS was consistent with the differential DNA methylation. Taken together, our data can be useful in deciphering the roles of DNA methylation in plant drought-tolerance variations and in emphasizing its function in alternative splicing.

## 1. Introduction

Maize (*Zea mays* L.) is an important crop with the highest yield among all cereal crops, and is used as food, feed, and fuel worldwide [1]. Abiotic stresses, especially drought, negatively affects maize production and even threatens global food security [2,3]. Drought tolerance, an intricate trait in plants, is associated with multiple genes and complex regulatory mechanisms. As “missing heritability”, the roles of epigenetic modifications in the drought response of maize need further investigation.

DNA methylation is a type of conserved epigenetic modification and plays an important role in the development [4], disease resistance [5], and response to abiotic stress [6] in plants. Four *Arabidopsis thaliana* populations showed a 30.0~78.3% enhancement of freezing tolerance after treatment with the DNA-methylation inhibitory reagent 5-azacytidine. DNA-methylation-related mutants, *cmt2*, *drm2* and *msh1*, displayed increased tolerance to heat stress and cold stress [7,8,9] and were associated with genome-wide changes in DNA methylation. Similarly, the disruption in the expression of genes involved in DNA methylation resulted in altered tolerance to abiotic stresses. Heterogenous expression of *AtROS1*, which is responsible for DNA demethylation, resulted in enhanced tolerance to salt stress in tobacco [10].

Compared with other abiotic stresses, the relationship between drought stress and DNA methylation changes is rather limited [6]. Nevertheless, there is considerable evidence indicating that drought stress can induce changes in DNA methylation. In wheat, multiple DNA methyltransferase genes were up-regulated under water stress [11]. Drought stress also induced genome-wide hyper-methylation in upland cotton and DNA methylation levels almost returned to their original levels after rewatering [12]. Rice cultivars with contrasting abiotic stress responses exhibit different DNA methylation and gene expression levels under stress [13,14]. Notably, the effects of drought stress on DNA methylation are to some extent cumulative and heritable [15], implying a strong association between DNA methylation and drought stress. More importantly, DNA methylation directly regulates the response to drought. In maize, DNA methylation, in the promoter of *NAC111,* resulted in increased drought sensitivity throughout repressed gene expression [16] and DNA methylation was found to be associated with the regulation of ABA-dependent gene expression [17]. However, the relationship between genome-wide DNA-methylation alteration and drought stress in maize remains unclear.

DNA methylation is usually implicated in abiotic stress throughout gene expression regulation. It is generally accepted that DNA methylation in the promoter region of genes inhibits gene expression by influencing the binding of transcription activators or repressors [18]. In contrast, gene-body DNA methylation (GbM), which is more prevalent and evolutionarily conserved, is still an enigmatic process [19,20]. Since the loss of GbM was reported unaccompanied by changes in gene expression [21], it was considered to be non-functional. Recently, GbM was reported to prevent cryptic transcription [22] or modulate alternative splicing (AS) [19,23,24,25], which suggests the roles of GbM are remarkably important and diverse. Thus, further investigation into how GbM functions in plant drought response is needed.

In our previous research, DNA methylation was found to be involved in the expressional regulation of natural antisense transcripts. However, the roles of DNA methylation in maize root drought response have not been further studied. In the present study, we reanalyzed the characteristics of DNA methylome in maize roots under well-watered (WW) and water stress (WS) conditions by using existing methylated DNA immunoprecipitation (MeDIP) sequencing data. The differentially methylated regions (DMRs) among maize inbred lines with extreme drought sensitivity and tolerance were investigated. We found that GbM was negatively correlated with gene expression and that exons with DNA methylation were preferentially spliced. The association between Small interfering RNA (siRNA) expression and the DNA methylation level highlights the important roles of the RdDM pathway under WS. Overall, these results expand our understanding of the underlying biological process and response regulation under drought stress in maize roots.

## 2. Results

### 2.1. Genome-Wide Distribution Patterns of DNA Methylation in Maize Roots

To investigate DNA methylation under different water conditions, MeDIP sequencing data from maize roots of drought-tolerant inbred lines (AC7643_DT and RIL208_DT) and drought-sensitive inbred lines (AC7729/TZSRW_DS and RIL64_DS) under both WW and WS conditions, were reanalyzed. We obtained 783,673,472 high-quality readings, with an average of 96.41% aligned to the maize reference genome Refv4 (Table S1). MACS2 was used to scan regions with highly enriched DNA methylation modification and a total of 405,687 DNA methylation peaks were identified in maize roots under both WW and WS conditions. To analyze the genome-wide DNA methylation landscapes, the distribution of those peaks along chromosomes was investigated. DNA methylation peaks were enriched in the pericentromeric regions and negatively correlated with gene distribution (Spearman’s rank correlation test, *p* < 0.001, *r* = −0.41, Figure S1).

Transposon elements (TEs) accounted for 81.62% of the DNA methylation peaks (Figure S2A), but the proportion of DNA-methylated TEs was lower than that of randomly selected regions (95.02%, χ^2^ test, *p* < 0.001). Among different classes of TEs, gypsy exhibited a distribution pattern with a significant positive correlation of DNA methylation peaks (Spearman’s rank correlation test, *p* < 0.001, *r* = 0.56). However, *copia* was preferentially located in chromosome arms and showed a significantly negative correlation with DNA methylation distribution (Spearman’s rank correlation test, *p* < 0.001, *r* = −0.48, Figure S1). Significantly fewer DNA methylation peaks (24.86%) were observed for *copia* than random segments (30.28%, χ^2^ test, *p* < 0.001, Figure S2B). DNA transposons were negatively correlated with DNA methylation peak genome-wide distribution (Spearman’s rank correlation test, *p* < 0.001, *helitron r* = −0.16, *TIR r* = −0.38), However, significantly more DNA methylation peaks were found for DNA transposons (*helitron*: 24.29%, *TIR*: 1.75%) than random segments (for *helitron*: 18.32%, for *TIR*: 0.50%, χ^2^ test, *p* < 0.001, Figure S2B).

**Figure 1:**
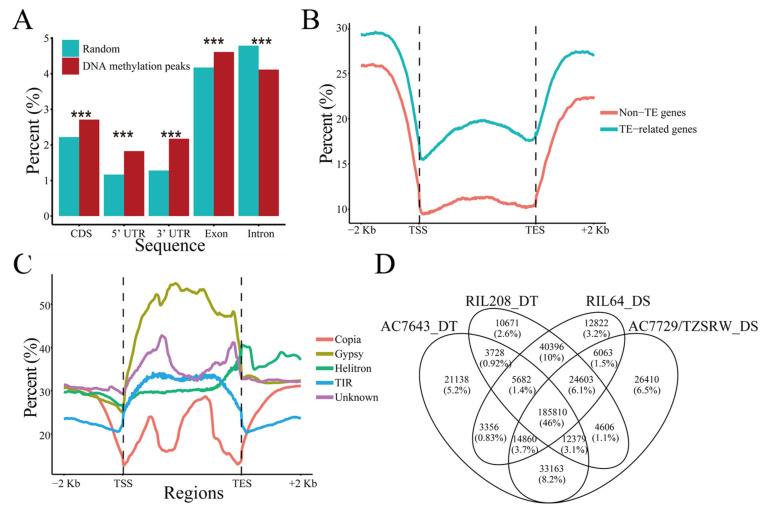
Summary of DNA methylation peaks. (**A**) The proportions of peaks in different genetic elements. The Y axis represents the proportions of random DNA segments and DNA methylation peaks in genetic elements. ***: *p* < 0.001, χ^2^ test. (**B**) The distribution pattern of DNA methylation peaks around the TSS (Transcription Start Site) and TES (Transcription End Site). Non-TE genes: genes without TE-overlapping; TE-related genes: genes overlapped TE. (**C**) The distribution of DNA methylation peaks around the TSS (TE Start Site) and TES (TE End Site) in different classes of TEs. (**D**) Venn diagram comparing DNA methylation peaks in four inbred lines.

Intergenic regions accounted for 80.92% of the entire genome, which contained significantly more DNA methylation peaks (86.38%, χ^2^ test, *p* < 0.001) than random segments (80.34%). The promoter harbored more DNA methylation peaks (TSS-2 kb: 7.40%; 2–5 kb: 9.67%) than downstream sequences of genes (TTS+2 kb: 6.57%; 2–5 kb: 7.63%. χ^2^ test, *p* < 0.001), suggesting the importance of DNA methylation in gene expression regulation. Only 6.26% of DNA methylation peaks were found in the gene body. Compared with introns, exons, including CDS and UTR, the gene body exhibited the enrichment of DNA methylation peaks (Figure 1A), which was in accordance with that observed in Arabidopsis [26] and rice [25].

The proportion of DNA-methylated genes with TE overlapping (TE-related genes, 45.79%) was significantly higher than that of genes without TE overlapping (non-TE genes, 29.84%, χ^2^ test, *p* < 0.001, Figure 1B), but the GC content of those genes was not consistent with this trend (Figure S3A). With respect to the distribution of DNA methylation along genetic elements, DNA methylation peaks were enriched in the promoter and downstream of genes. In contrast, DNA methylation in the flanking region of TE was significantly lower than it was in the TE (Figure S3B,C). Intriguingly, DNA methylation in different classes of TE exhibited unique distribution characteristics. The DNA methylation peaks enriched in the bodies of *gypsy* and *TIR*, but downstream of *helitron* (Figure 1C), were independent of GC content (Figure S3D).

### 2.2. Inheritance of DNA Methylation Modifications Increased under Drought Stress

We found that 88.62% of DNA methylation peaks were shared among at least two samples and 11.38% of all peaks existed in only one sample (Figure 1D), indicating that DNA methylation was to some extent conserved. The number of DNA methylation peaks specifically identified under WW condition (7.55%) was significantly higher than that under WS condition (5.82%, χ^2^ test, *p* < 0.001, Figure S4A). The drought-tolerant inbred lines had more specific peaks (11.17%) than the drought-sensitive inbred lines (8.76%, χ^2^ test, *p* < 0.001, Figure S4B). In addition, a higher percentage of specific peaks was identified in the parental lines (19.89%) than in the derived RILs (15.75%, χ^2^ test, *p* < 0.001, Figure S4C). The number of common DNA methylation peaks under different water conditions was higher than that between inbred lines with various drought sensitivities (WW and WS: 86.63%, the drought-tolerant inbred lines and the drought-sensitive lines: 80.08%, Figure S4A,B). The result indicated that variations in DNA methylation in different genetic backgrounds were larger than those under different water regimes. It is worth noting that the proportion of DNA methylation peaks shared by the parental lines and RILs was significantly lower than the proportion shared by the former two combinations (64.36%, χ^2^ test, *p* < 0.001, Figure S4C). The proportion of DNA methylation peaks shared by the RILs (64.07%) was significantly higher than that shared by the parental lines (57.57%, χ^2^ test, *p* < 0.001, Figure S4D,E). Furthermore, 76.39% of the DNA methylation peaks identified in the parental lines also existed in the RILs. These results suggested that DNA methylation may be inherited and that recombination is involved in this inheritance.

**Figure 2:**
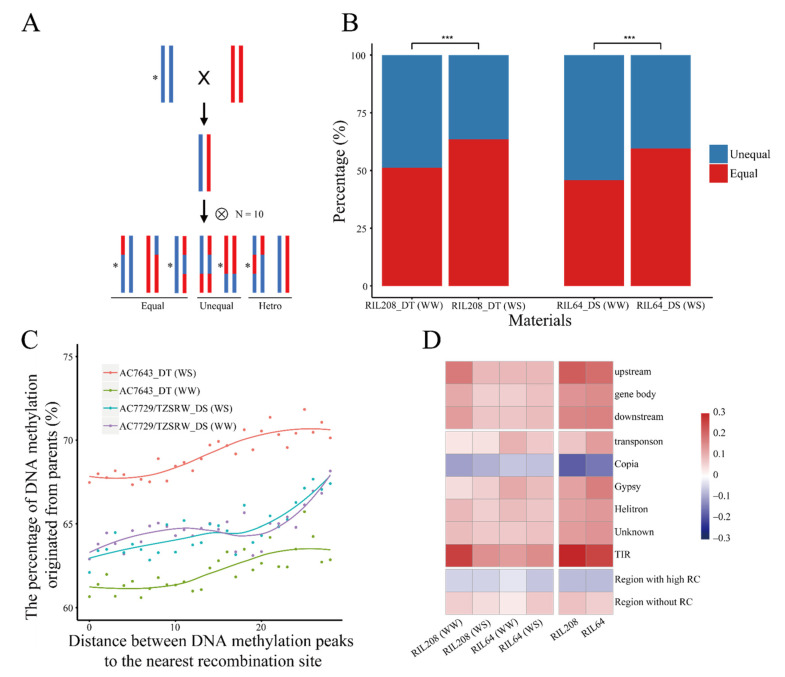
The inheritance of DNA methylation. (**A**) The diagram of the DNA methylation relationship of the RILs and sequence-origin parental lines. The blue and red bars mean DNA sequences originated from the two parental lines, respectively. The asterisk represents the existence of DNA methylation peaks. (**B**) The histogram of the proportion of DNA methylation peaks in RILs consistent with those of the corresponding parental line. WW: Well Water, WS: Water Stress. ***: *p* < 0.001, χ^2^ test. (**C**) The xy-plot of the proportion of DNA methylation peaks in RILs consistent with that of the parental lines and the distance to the nearest recombination site. (**D**) The heatmap of the conservation of DNA methylation in different regions. Data was normalized by random DNA segments of length equal to DNA methylation peaks. RC: Recombination.

To further analyze the inheritance of DNA methylation, single nucleotide polymorphisms (SNPs) obtained by genotyping-by-sequencing in the RIL population (n = 193) were used to construct a bin map and to calculate recombination ratio (Figure S5A). The vast majority of SNPs (93.87%) obtained from the RNA-Seq dataset of RIL208_DT and RIL64_DS were concordant with the genetic bins, confirming the reliability of the bin map (Figure S5B). The consistency of the DNA methylation status between RILs and the corresponding sequence-origin parental lines was estimated using the index of inheritance as shown in Figure 2A. We found that the DNA methylation status of RILs was more likely to be in accordance with the corresponding parental line (the proportion of concordant DNA methylation status between RIL208_DT and its sequence-origin parental lines: 77.25%, RIL64_DS: 78.00%) than that of the other parental line (RIL208_DT: 74.78%, RIL64_DS: 77.26%, Figure S5C). More interestingly, DNA methylation regions detected under WS were preferentially inherited from the corresponding sequence-origin parental lines, indicating the strong inheritance of DNA methylation modifications related to drought stress (Figure 2B).

We also analyzed the pattern of inheritance of DNA methylation in different genic elements. As expected, recombination was closely associated with DNA methylation inheritance. The relationship between the distance from the DNA methylation site to the nearest recombination site and the percentage of DNA methylation originating from parents was further analyzed. The DNA methylation peaks closer to the recombination sites were likely to be less consistent with the sequence-origin parental lines (Figure 2C) and the inheritance of DNA methylation in regions without recombination events was higher than that in recombination “hotspots” (χ^2^ test, *p* < 0.001, Figure 2D). Notably, the inheritance of DNA methylation in different genic regions was different. Except for *copia*, genes and most TEs showed higher consistency than those of the random sequences (χ^2^ test, *p* < 0.001, Figure 2D).

### 2.3. DNA Methylation Varied in Different Genotypes and Genomic Regions under Drought Stress

**Figure 3:**
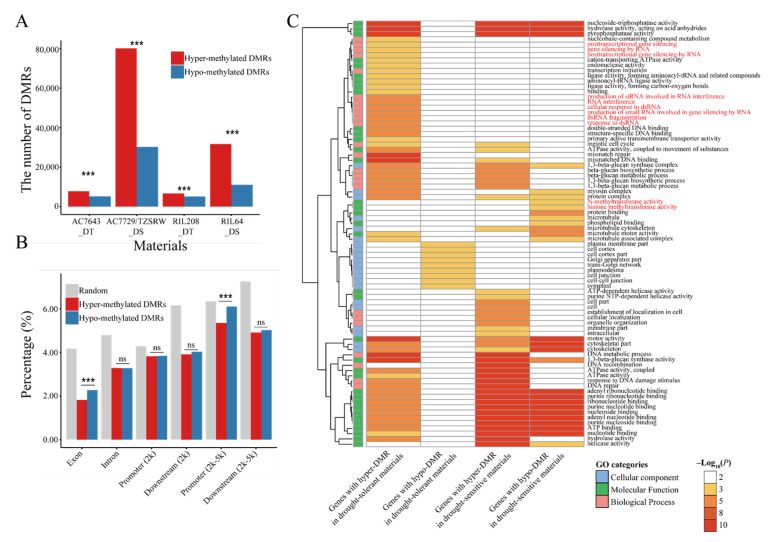
Summary of differentially methylated regions (DMRs). (**A**) The number of DMRs in four inbred lines. Hyper-DMRs mean regions where DNA methylation levels increased under WS, while hypo-methylated DMRs stand for regions of decreased DNA methylation levels under WS. ***: *p* < 0.001, χ^2^ test. (**B**) The proportions of DMRs located in different genetic elements. ***: *p* < 0.001, ns: not significant, χ^2^ test. (**C**) Gene Ontology (GO) enrichment terms of genes with DMRs in gene body. Genes involved in establishment and maintenance of DNA methylation were highlighted by red color.

To analyze the response of DNA methylation under WS, we compared DNA methylation modification levels under different water regimes by MACS2. Compared to WW condition, 172,500 DMRs were found to have distinct DNA methylation statuses in four inbred lines under WS. The number of regions with increased DNA methylation levels under WS condition (hyper-DMRs, 121,405) were significantly higher than those for regions with decreased DNA methylation levels under WS condition (hypo-DMRs, 48,823. χ^2^ test, *p* < 0.001). We observed an inconsistent trend in DNA methylation in inbred lines with different drought sensitivities. DNA methylation of the drought-sensitive inbred lines was more sensitive to drought stress. The linesAC7729/TZSRW_DS (110,437) and RIL64_DS (42,757) had many more regions that exhibited significant responses than the drought-tolerant inbred lines (AC7643_DT: 13,048; RIL208_DT: 11,854. χ^2^ test, *p* < 0.001). The response of DNA methylation in the drought-sensitive inbred lines increased under drought stress (AC7729/TZSRW_DS: 72.60%; RIL64_DS: 74.16%), while the numbers of hyper-DMRs and hypo-DMRs were roughly comparable in drought-tolerant inbred lines (Figure 3A). Remarkably, a comparison of DMRs among different inbred lines revealed that most of the DMRs were genotype specific. Only 3.19% of DMRs were found in more than one material (Figure S6), which was consistent with previously reported results in rice [14].

Given that DNA methylation may be involved in drought response through gene regulation, we counted the distribution of DMRs in genic regions. The proportion of DMRs located in the gene body or promoter was significantly lower than that of random segments (Figure 3B), suggesting that DNA methylation associated with genes was relatively stable, which was consistent with the findings in plants and animals [19]. In addition, the proportion of exons (2.27%) or gene promoters (6.10%) with hypo-DMRs was significantly higher than that of exons (1.82%, χ^2^ test, *p* < 0.001) or promoters (5.35%, χ^2^ test, *p* < 0.001) with hyper-DMRs (Figure 3B), emphasizing the specific function of DNA methylation in promoters and exons under WS.

As DNA methylation in TEs was an important regulatory factor, DNA methylation change in TEs under WS was evaluated. Most of the DMRs (77.45%) were located in TEs and various classes of TEs showed differential responses to DNA methylation. *Gypsy* was enriched with DMRs (DMRs located in gypsy: 64.09%; random DNA segments in gypsy: 50.42%, χ^2^ test, *p* < 0.001), whereas *copia* had fewer DMRs (20.36% vs. 30.28%, χ^2^ test, *p* < 0.001, Figure S7). Furthermore, there was also material specific for the DNA methylation response in TE. The drought-sensitive inbred lines enriched DMRs associated with gypsy (68.80%), whereas the proportion of hyper-methylated DMRs associated with *copia* was significantly lower (19.59%; mean of others: 21.76%, χ^2^ test, *p* < 0.001). In addition to the hypo-methylated DMR in the promoter, DMRs were always enriched in TE-related genes. (χ^2^ test, *p* < 0.001, Table 1).

To analyze the possible distinct functions of differential DNA methylation responses in four inbred lines, Gene Ontology (GO) analysis of genes with DMRs in the promoters and the gene body was performed, in drought-tolerant inbred lines and drought-sensitive inbred lines, respectively. Unexpectedly, there was no molecular function terms in the GO enrichment results of genes with DMRs in the promoters (Table S2) and several diverse GO terms were enriched in genes with DMRs in the gene body. The GO terms ATP binding (GO:0005524), hydrolase activity (GO:0016787), and pyrophosphatase activity (GO:0016462) were enriched in genes with hyper- or hypo-DMRs in both drought-sensitivity lines (Figure 3C). However, production of a small number of RNA involved in gene silencing by RNA (GO:0070918) and RNA interference (GO:0016246) were only enriched in genes with hyper-DMRs in the drought-tolerant inbred lines. Similarly, histone methyltransferase activity (GO:0042054) was only enriched in genes with hypo-DMRs in the drought-sensitive inbred lines. These discrepancies between DNA methylation alterations under WS condition in different inbred lines implied that inbred lines with different drought sensitivities may respond to drought stress in different ways.

### 2.4. Potential Role of DNA Methylation in Drought Stress Response Regulation

**Figure 4:**
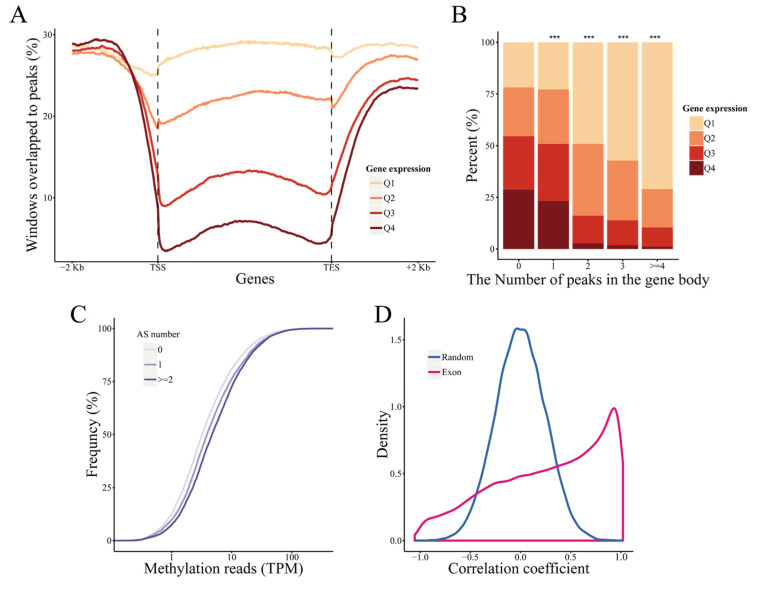
DNA methylation was related to gene expression and alternative splicing. (**A**) The proportion of DNA methylation peaks are plotted along the gene region as well as the upstream (-2 kb) and downstream (+2 kb) of genes with different expression levels. (**B**) The expression pattern of genes with varying number of DNA methylation peaks in gene body. ***: *p* < 0.001, χ^2^ test. (**C**) The DNA methylation levels of genes with varying alternative splicing (AS) events number. (**D**) The distribution of correlation coefficients between the expression and DNA methylation level of exon in genes with multiple exons.

To further clarify the role of differential DNA methylation changes in maize’s response to drought stress, we investigated whether DNA methylation had the ability to regulate gene expression under WW and WS conditions. First, we divided the total number of genes into four groups by the quantile of expression level and the DNA methylation levels of these genes were compared. DNA methylation in the promoter is generally considered to result in the repression of gene expression. However, DNA methylation in promoter and gene expression levels showed a weakly positive correlation (Spearman’s rank correlation test, *p* < 0.001, *r* = 0.02). In contrast, a significantly negative correlation between DNA methylation and gene expression was detected in the gene body (Spearman’s rank correlation test, *p* < 0.001, *r* = −0.31). As shown in Figure 4A, genes with a low expression level usually showed higher DNA methylation levels than genes with a high expression level (Student’s *t* test, *p* < 0.001). To further investigate the extent of the effect of DNA methylation on gene expression, the expression levels of genes with different DNA methylation levels in the gene body were evaluated. The low DNA methylation group (without DNA methylation peaks in gene body) consisted of a similar number of lowly to highly expressed genes, whereas highly expressed genes were almost depleted in the high DNA methylation group (with at least four DNA methylation peaks in the gene body, Figure 4B). In addition, gene expression showed similar distributions among the four inbred lines (Figure S8A), whereas DNA methylation levels exhibited differences. DNA methylation levels in the gene body of lowly expressed genes in WW or drought-sensitive inbred lines were always higher than those of genes in WS or drought-tolerant inbred lines (Student’s *t* test, *p* < 0.001, Figure S8B).

Except for gene expression regulation, DNA methylation in the gene body may affect gene AS response to drought, and thus we investigated the relationship between DNA methylation and AS. First, we depicted the DNA methylation coverage around the exon junction, but did not find any evident signals (Figure S9A). Next, we chose genes with equal expression at random as negative controls (Figure S9B) and compared the DNA methylation level and AS number. The DNA methylation level of genes with more AS events was significantly higher than that of genes without AS events (Student’s *t* test, *p* < 0.001, Figure 4C). Moreover, by comparing the DNA methylation level of genes with different expression levels and determining the correlation, we found a significant positive correlation between DNA methylation and the expression level of exons (Spearman’s rank correlation test, *p* < 0.001, *r* = 0.18, Figure S9C). Approximately 60% of correlation coefficients in genes with multiple exons were above zero, which was significantly higher than that of the permutation test (Figure 4D). Furthermore, AS can result in introns being retained in mature mRNA, so we compared the relationship between retained introns and DNA methylation. The percent spliced in (PSI) of retained introns with DNA methylation was significantly higher than that of retained introns without DNA methylation peaks (Student’s *t* test, *p* < 0.001, Figure S9D), which implied that DNA methylation in the gene body was closely related to exon splicing.

Given the typical DNA methylation characteristics, TE may affect the expression of nearby genes and the functioning of DNA methylation response to drought stress. Therefore, we compared the expression level of genes with TE overlapping and genes without TE overlapping. Different classes of TEs showed distinct patterns in terms of the effect on gene expression. The expression levels of genes overlapping *copia*, *gypsy*, or *helitron* was significantly lower than those of genes without TE overlapping (Student’s *t* test, *p* < 0.001), whereas the expression level of genes overlapping *TIR* was significantly higher (Student’s *t* test, *p* < 0.001, Figure S10A). We then analyzed the effect of DNA methylation in different TEs on gene expression. As expected, the DNA methylation of TE resulted in a decrease in the expression of the nearby gene. However, the expression of genes with *TIR* was not affected by DNA methylation (Figure S10B). To confirm this result, we further compared DNA methylation levels in genes with their equivalent expression levels. In each expression group, genes with overlapping TEs of the *copia*, *gypsy* and *helitron* classes tended to be highly methylated than those without TEs, and genes with more methylated TEs (except for *TIR* TEs) tended to show lower levels of expression than genes with less methylated TEs (Figure S10C).

### 2.5. DNA Methylation Regulates Gene Expression and Alternative Splicing in Response to Drought Stress

**Figure 5:**
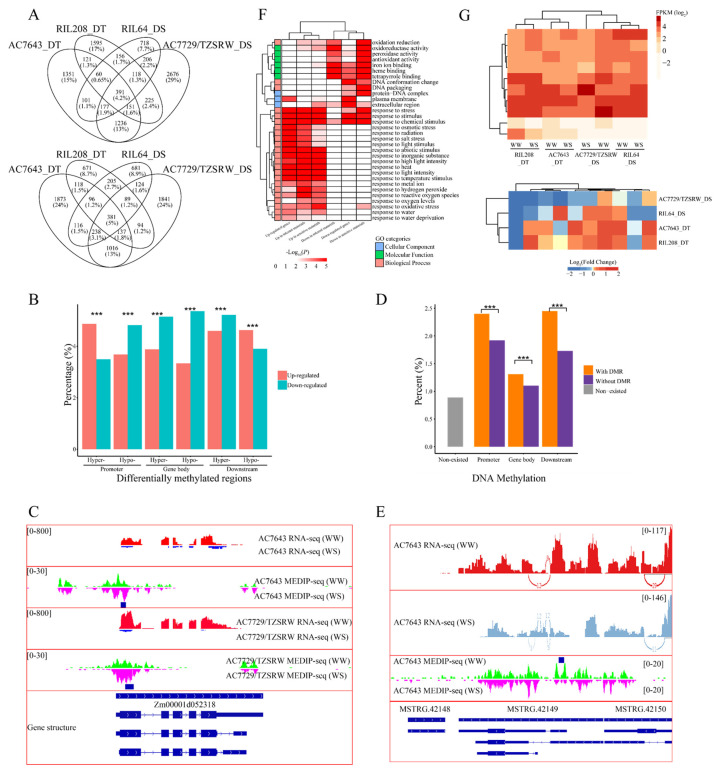
DNA methylation involved in gene response under WS. (**A**) Venn diagram comparing up-regulated genes (upper) and down-regulated genes (bottom) in four inbred lines. (**B**) The proportion of up-regulation and down-regulation genes with different DNA methylation response in the promoter, the gene body and downstream. ***: *p* < 0.001, χ^2^ test. (**C**) Examples of genes with hyper-methylated in the gene body and decreased expression under WS. (**D**) The proportion of genes with DAS under water stress. ***: *p* < 0.001, χ^2^ test. (**E**) Examples of genes hyper-methylated in the gene body and differential AS pattern under WS. (**F**) GO enrichment of differentially expressed genes (DEGs). Only GO terms with FDR < 0.001 in at least two groups were plotted. (**G**) The clustered heatmap of expression (upper) and fold change (bottom) under WS of genes which was association with DMRs and classified in “gene silencing by RNA” GO term.

To elucidate whether the ability of DNA methylation in regulating gene expression and AS was used in response to drought stress, we compared the change in the gen expression level, splicing pattern and DNA methylation under WS. The process of edgeR was used to identify the WS-responsive genes, and we found that an average of 13.73% of all genes responded to drought. Unlike the significant difference in DMRs, the number of up- and down-regulated genes was roughly equivalent; 7.38% and 6.35% of the genes were up- and down-regulated under WS, respectively. Approximately 5% of differentially expressed genes (DEGs) were common to all materials. The intersect set of up-regulated genes between the two RIL offsprings (725 of 4019, 18.04%) was significantly less than that between the two parental lines (1955 of 6813, 28.70%, χ^2^ test, *p* < 0.001), although the genetic distance between the parent inbred lines was, theoretically, higher than the genetic distance between the two RIL offsprings (Figure 5A). Therefore, TEs may affect the response of nearby genes. The proportion of down-regulated genes with overlapped TEs under WS (6.08%) was significantly lower than that of genes without TE overlapping (7.04%, χ^2^ test, *p* < 0.001, Table 2), especially in *copia* (5.46% vs. 6.34%, χ^2^ test, *p* < 0.001).

To further analyze the function of DNA methylation in the regulation of gene expression under drought stress, we compared the response of genes and DMR. DNA methylation under WS did not change the proportion of nearby genes that significantly responded to drought stress, however, we found consistency in the response of gene expression with DNA methylation in the promoters. As shown in Figure 5B, genes with increased DNA methylation levels in the promoter were more up-regulated under WS (up-regulated: 4.87% vs. down-regulated: 3.68%, χ^2^ test, *p* < 0.001). In contrast, genes with hypo-methylated regions in the promoter were more down-regulated (down-regulated: 4.82% vs. up-regulated: 3.50%, χ^2^ test, *p* < 0.001), which indicated that DNA methylation was indeed involved in the response of gene expression under drought stress. Unexpectedly, the changes in gene expression under WS were discordant with the DNA methylation level altered in the gene body. In both genes with increased DNA methylation levels and genes with hypo-DMR, there were always more down- than up-regulated genes (3.88% vs. 3.33%, χ^2^ test, *p* < 0.001). For example, Zm00001d052318, the ortholog of *ATTBL34* in maize, was down-regulated under WS while the DNA methylation level in the gene body increased under WS (Figure 5C).

To study the association between the change in DNA methylation and AS under WS, we compared the AS patterns and DMRs. Genes with altered DNA methylation under WS had a significantly higher proportion of differential AS (DAS, 2.40% in the promoter, 1.31% in the gene body, and 2.45% in the downstream region) than genes without DMR (0.89%, χ^2^ test, *p* < 0.001). Furthermore, genes with DMR always had a higher ratio of DAS than genes with stable DNA methylation under WS. As shown in Figure 5E, MSTRG.42149 simultaneously undergoes AS and significant DNA methylation responses under WS.

To further analyze the function of WS-responsive genes, enrichment analysis was used to explore the GO annotation of these genes. We found that there were different GO enrichment terms in WS-responsive genes in inbred lines with different drought sensitivities. As shown in Figure 5F, the up- and down-regulated genes had a few common GO terms, including response to stress (GO:0006950) and stimulus (GO:0050896). The GO terms of response to abiotic stresses, including water (GO:0009415), salt (GO:0009651), metal ion (GO:0010038), light (GO:0009416), temperature (GO:0009266), and osmotic stress (GO:0006970), were only enriched in the up-regulated genes; whereas the down-regulated genes enriched oxidoreductase activity (GO:0016491), heme binding (GO:0020037), and iron ion binding (GO:0005506). Surprisingly, the GO terms DNA conformation change (GO:0071103), protein-DNA complex (GO:0032993), and DNA packaging (GO:0006323), had close associations with DNA methylation and histone modification and were only enriched in the drought-sensitive inbred lines compared with those in the drought-tolerant lines. It is noteworthy that the expression clustering of genes associated with the GO term gene silencing by RNA (GO:0031047), which was enriched in genes with DMR, was consistent with the drought sensitivities of inbred lines (Figure 5G). Under WS, these genes mainly increased their expression in the drought-tolerant inbred lines, but were down-regulated in the drought-sensitive inbred lines (Figure 5G). We also analyzed the response of several genes involved in regulating DNA methylation. In Figure S11A, *DRM2* (Zm00001d044187), which was responsible for the establishment and maintenance of DNA methylation, showed the material specificity in response to drought stress. However, *DDM1A* (Zm00001d007978) and *DDM1B* (Zm00001d033827), which were required for maintaining DNA methylation, were down-regulated in AC7643_DT and AC7729/TZSRW_DS. Combined with the occupancy of *DDM1* identified by CHIP experiments with specific antibodies [27], we analyzed the DNA methylation responses of *DDM1*-occupied genes. In AC7643_DT and AC7729/TZSRW_DS, the proportion of differential DNA methylation that occurred in *DDM1*-occupied genes was significantly higher than that of total genes. Surprisingly, the proportion of hyper- and hypo-methylated genes was significantly higher, although *DDM1* was down-regulated under water stress (Figure S11B).

### 2.6. The Stress Response of DNA Methylation Is Significantly Correlated with siRNA Expression

**Figure 6:**
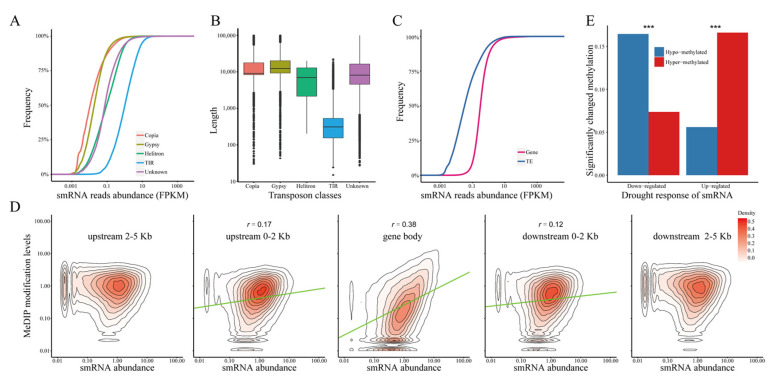
The closed relationship between small interfering RNA (siRNA) expression and DNA methylation. The distribution of siRNA expression (**A**) and length (**B**) among varying classes of TEs. (**C**) The expression level of siRNA in genes and TEs. (**D**) The correlation of siRNA expression (*x*-axis) and DNA methylation level (*y*-axis) in different regions. The green lines fit the regression between siRNA abundance and DNA methylation level by genear linear model. (**E**) The proportion of up-/down-regulated siRNA in hyper-DMRs or hypo-DMRs under WS. ***: *p* < 0.001, χ^2^ test.

The discordance between DNA methylation and gene expression suggested that this regulation was more complex than expected. To analyze the preferential regulation of DNA methylation in response to drought stress, it was important to compare the changes in upstream regulators under WS. Considering that siRNAs play an important role in a variety of biological processes such as de novo DNA methylation, TE repression, and gene regulation, we selected siRNA to identify their association under different water regimes. First, we compared siRNA expression at different genomic locations. DNA TEs (*helitron* and *TIR*) showed higher siRNA expression than LTR TEs (*copia* and *gypsy*, Student’s *t* test, *p* < 0.001, Figure 6A), which were probably related to TE length (Figure 6B). Unexpectedly, the abundance of siRNAs in TEs was significantly lower than that in the genes (Student’s *t* test, *p* < 0.001, Figure 6C), suggesting that siRNA might have a strong association with genes.

We then analyzed the correlation coefficient between siRNA expression and DNA methylation levels in maize roots. The correlation coefficients in the gene body were 0.38 (Spearman’s rank correlation test, *p* < 0.001). Surprisingly, in the flanking region of the gene body, the correlation decreased in a 2 kb flanking region of gene (correlation coefficients: 0.17 and 0.12, respectively, Spearman’s rank correlation test, *p* < 0.001) and destroyed the region that was 2–5 kb away from the gene body (Spearman’s rank correlation test, *p* > 0.05, Figure 6D), re-emphasizing the importance of GbM. In addition, exon showed a weak correlation (correlation coefficients: 0.26, Spearman’s rank correlation test, *p* < 0.001) and intron showed a much stronger positive correlation (correlation coefficients: 0.46, Spearman’s rank correlation test, *p* < 0.001). There were varying degrees of correlation in the different classes of TEs. The correlation coefficient between siRNA expression and the DNA methylation modification level in copia (0.52) was the highest, whereas that in gypsy it was the lowest (0.28).

Furthermore, we compared the response of siRNA in DMRs under WS. As shown in Figure 6E, the proportion of up-regulated siRNAs in the hyper-methylated region under WS (16.60%) was significantly higher than that of the down-regulated siRNAs (7.38%, χ^2^ test, *p* < 0.001), and hypo-DMRs had fewer up-regulated siRNAs than the down-regulated siRNAs under WS (5.60% vs. 16.45%, χ^2^ test, *p* < 0.001). These results illustrated their close relationship in maize roots, and implied that the response of DNA methylation under drought stress may be precisely regulated by siRNA.

## 3. Discussion

DNA methylation is an important epigenetic modification and it has been widely implicated in plant development and stress responses [28,29,30]. In this study, we repurposed generated MeDIP-Seq data to profile the DNA methylation map of four inbred lines with different drought sensitivities under WW and WS conditions. The results illustrated that there were distinct drought stress responses of DNA methylation among drought-tolerant and drought-sensitive inbred lines, and DNA methylation, in response to drought stress, is involved in not only gene expression inhibition but also alternative splicing.

Studies in many plants have shown genome-wide changes in DNA methylome and the alteration of DNA methylation in some stress-responsive genes [14,31]. However, whether there is causal relationship between DNA methylation alteration and abiotic stress, is still unclear. Considering that DNA methylation can regulate gene expression [18], alterations in DNA methylation under abiotic stress are generally thought to be responsible for changes in gene expression levels [32]. However, there is another hypothesis that the abiotic stress decreased stability in the DNA methylome resulting in higher DNA methylation variation in response to abiotic stress [33]. One proof is that the stress-induced DNA methylation changes occurred after the alteration of nearby gene expression under phosphate starvation [30], implying that there may be no causal relationship between DNA methylation changes and stress-responsive gene expression. In our study, DMRs were not randomly distributed and exhibited unique patterns compared to DNA methylation distribution. Moreover, siRNA could act as a possible upstream regulator of DNA methylation [34] and had a consistent trend with DNA methylation in response to stress, suggesting that the response of DNA methylation was more likely to be precisely controlled. We also found that genes with DNA methylation alterations under WS enriched GO temrs related to the establishment and maintenance of DNA methylatio, such as the production of small RNA involved in gene silencing by RNA and histone methyltransferase activitye. Small RNA plays an important role in the RdDM pathway, which mediates *de novo* DNA methylation in plants [18,34]. Multiple lines of evidence have demonstrated the interplay between DNA methylation and histone modification, such as CHG methylation by CMT3 depending on H3K9me2 [35]. These possible feedback mechanisms that can fine-tune DNA methylation may account for the reverse time order of DNA methylation and changes in gene expression levels. Overall, our data strongly support the judgement that DNA methylation changes are a cause, not a consequence, of drought stress response.

DNA methylation in maize can be heritable [36,37] and changes under stress may influence subsequent generations [38]. We also found that the DNA methylation status in the offspring was more similar to that of the sequence-origin parents and the inheritance increased under drought stress, highlighting the potential function of DNA methylation. Based on this hypothesis, multiple projects were implemented for mining the differences in DNA methylation association with phenotype traits in large-scale populations, such as local adaptation [39] and metabolic traits [40]. By comparing responses under WS, we found that inbred lines with different drought sensitivities exhibited distinct stability of DNA methylome, which is similar to the findings of previous studies in rice [14,15]. Especially, the number of regions with increased and decreased DNA methylation levels was roughly equivalent in drought-tolerant lines but had significantly more hyper-DMRs in drought-sensitive lines under WS. Furthermore, the distinct DNA methylation alteration and differential expression of some regulatory genes implied that different regulatory mechanisms existed in genotype-specific inbred lines. These results indicated that DNA methylation was involved in the regulation of drought stress response by adjusting the expression of stress-induced genes, which contributed to the variation in drought tolerance among maize inbred lines. However, the mechanisms involved remain unclear. There have been several reports on the analysis of the genetic basis of DNA methylation variation [40,41], and further use of these data to mine the relationship between epigenetic modification and abiotic stress response is needed.

DNA methylation regulates gene expression, RNA processing, and TE silencing by defined chromatin structure and accessibility [18]. The way in which DNA methylation responds to abiotic stresses should also be relevant to these molecular functions. We found that DNA methylation in the promoter was positively associated with gene expression and was a concordant trend of alteration in DNA methylation in the promoter and gene response under WS condition, in agreement with a study on rice [31]. The positive correlation between DNA methylation and gene expression levels was also reported in several genes for functional studies, such as the expression of *ROS1* being fine-tuned by DNA methylation of MEMS in the promoter [42] and the SUVH proteins functioning as a DNA methylation reader and enhancing gene transcription by recruiting the DNAJ proteins in Arabidopsis [43]. These results were discordant with the general consensus that DNA methylation in the promoter inhibits gene expression [44,45] and may be related to the binding of some specific transcription repressors and activators in maize roots. Meanwhile, DNA methylation in the gene body was negatively correlated with gene expression, which is consistent with the findings of previous studies in Arabidopsis [26] and cotton [46]. However, the loss of GbM did not result in changes in gene expression [21], and we also found that changes in gene expression and DNA methylation under drought stress were inconsistent. Therefore, the roles of DNA methylation in gene expression regulation need further study.

Furthermore, there was a significant positive correlation between DNA methylation and exon splicing and increased DNA methylation in the retained introns, suggesting that DNA methylation in the gene body can enhance splicing efficiency at the genome-wide level, which corresponds with findings in human [23] and rice [25] about the ability of DNA methylation to regulate AS. A new method that manipulates DNA methylation in a site-specific manner by dCas9 was implemented to test the importance of GbM on AS. To our knowledge, this is the first direct evidence that DNA methylation altered inclusive levels of alternatively spliced exons, and our data suggested that this phenomenon may be pervasive in plants and stress response.

TEs are an important part of the plant genome, and TE silencing by DNA methylation is of great importance. The gene NMR19-4, located in the promoter regions of pheophytin pheophorbide hydrolase (PPH), negatively regulates PPH expression and leads to a variation in leaf senescence [47]. An 82-bp MITE inserts ZmNAC111 into the promoter and represses gene expression via RNA-directed DNA methylation, resulting in increased drought sensitivity [16]. In our study, DNA methylation modification, variation under drought stress, effects on nearby gene expression levels of different classes of TEs, and the correlations with siRNA abundance all showed distinct patterns, highlighting functional differences in TEs. However, only a few previous studies have reported that DNA methylation in specific TE families is associated with distinct functions [48,49], and information regarding this is still limited. The DNA methylation characteristics of these TEs should be fully considered in subsequent studies.

The development of high-throughput sequencing technology has provided an opportunity to profile genome-wide DNA methylation. In this study, we identified genome-wide DNA methylation in different drought sensitivities lines. However, due to limited resolution and population size, we could not accurately identify DNA methylation sites associated with drought sensitivity variation. Genome-wide bisulfite sequencing can be used to evaluate DNA methylation differences and responses at a single-nucleotide resolution. Combining the reported DNA methylation profile in large-scale populations and gene expression or phenotype data under abiotic stress, mining the epigenetic basis of these traits will be the focus of further studies. The use of genome directional editing technologies to directly manipulate DNA methylation modification for functional analysis is also an important way to understand how DNA methylation is involved in abiotic stress response.

## 4. Conclusions

Increased DNA methylation levels were observed under WS and the methylome of drought-tolerant inbred lines were much stable than theose of drought-sensitive inbred lines. Distinctive differentially methylated genes among diverse genetic backgrounds suggested that DNA methylation is one of the reasons accounting for their drought tolerance variations. The inheritance of DNA methylation was coupled with the genetic inheritance. DNA methylation in the gene body showed a negative correlation with gene expression but a positive correlation with exon-splicing events, indicating the response of DNA methylation to drought stress is not only in gene expression inhibition but also alternative splicing. The response of endogenous siRNAs under WS may lead to differential DNA methylation.

## 5. Materials and Methods

### 5.1. DNA Methylation Peak Calling

MeDIP sequencing data of the roots of two extreme drought-tolerant inbred lines (AC7643_DT and the derivative RIL208_DT) and two extreme drought-sensitive inbred lines (AC7729/TZSRW_DS and the derivative RIL64_DS) were used for DNA methylation analysis. The data were generated under WS and WW conditions in our previous study and were obtained from the NCBI SRA database (accession number: SRP063383) [50].

High-quality reads were mapped to the maize B73 reference v4 (Refv4) genome using Bowtie2 (ver. 2.2.9) with default parameters and the best-matched reads were used in the downstream analysis. The MACS2 (ver. 2.2.9) [51] software (download date: 25 February 2017) Xiaole Shirley Liu, Cambridge, MA, USA) was used to search for highly significant enriched DNA methylation regions with *-m 10 100000*, regions with corrected *p*-value < 0.05 were referred to as DNA methylation peaks. To estimate genome-wide DNA methylation, average peaks, genes and TE coverage were calculated in 1-Mb sliding windows. Reference genes and TE annotations were downloaded from ftp://ftp.ensemblgenomes.org/pub/plants/release-35/gff3/zea_mays/Zea_mays.AGPv4.35.chr.gff3.gz (accessed on 2 April 2017) and ftp://ftp.gramene.org/pub/gramene/release-/gff3/zea_mays/repeat_annotation/B73v4.TE.filtered.gff3.gz (accessed on 2 April 2017), respectively. Genetic elements overlapping DNA methylation peaks were identified using GenomicRanges package (ver. 1.34.0, download date: 28 December 2018, Michael Lawrence, South San Francisco, CA, USA) in R. DNA segments of length equal to DNA methylation peaks were selected randomly as controls, and the chi-square (χ^2^) test was used to compare the distribution of DNA methylation peaks in various genetic elements. The average coverage of DNA methylation peaks in 2 kb flanking regions of genes or TEs was calculated in 100-bp windows with a 20-bp step.

### 5.2. Construction and Validation of Bin Map

Genotyping-by-sequencing data of the RILs and parental lines (NCBI accession: PRJNA597789) were used to call genotypes using the TASSEL-GBS pipeline (ver. 5.2.51, download date: 13 December 2019, Jeffrey C. Glaubitz, Ithaca, NY, USA) with maize B73 Refv4 as the reference genome. After filtering the missing ratio of > 0.05, SNPs differing between the two parental lines, AC7643_DT and AC7729/TZSRW_DS, were used to construct genomic bins using SNPbinner (ver. 0.1.4, download date: 27 April 2019, Itay Gonda, Davis, CA, USA) [52] with a minimum length of 1000 bp. For validation of bins, RNA-Seq data were used for SNP calling. After aligning reads to the maize B73 Refv4 genome using bwa (ver. 0.7.17, download date: 1 April 2016, Heng Li, Cambridge, CB10 1SA, UK) with default settings, Picard (ver. 2.15.0, download date: 7 July 2019, Broad Institute, Cambridge, MA, USA) was used to mark duplicated reads and sort bam with default settings. GATK (ver. 3.8, download date: 7 July 2019, Broad Institute, Cambridge, MA, USA) was used for variant calling and joint genotyping as previously described [53], and SNPs were filtered as follows: mapping read quality > 40, quality > 20 and depth > 10. To analyze the relationship between DNA methylation and recombination, a recombination event was defined as different adjacent bins and recombination events were counted in per Mb windows.

### 5.3. Differentially Methylated Region Calling

The software MACS2 was used to identify DMRs by comparing the number of methylated reads under WW and WS conditions. DMRs were integrated by genomic location, and UpSetR (ver. 1.3.3, download date: 30 June 2019, Jake R Conway, Boston, MA, USA) was used to depict the number of intersect sets of DMRs between the four inbred lines. Genes with hyper- or hypo-DMRs in inbred lines with different drought sensitivities were used in the GO enrichment analysis using agriGO v2 (http://systemsbiology.cau.edu.cn/agriGOv2/classification_analysis.php?category=Plant&&family=Poaceae) (accessed on 17 June 2019) [54] with maize locus ID v3.30 as reference.

### 5.4. Identification of Differentially Expressed Genes

Maize RNA-Seq data were downloaded from the NCBI SRA database (accession number: SRP063383) and aligned to the maize reference genome (Refv4) using HISAT2 (ver. 2.1.0, download date: 8 June 2017, Daehwan Kim, Dallas, TX, USA) with *--max-intronlen 2000* guided by reference-gene annotation. The sorted bam file of each sample was used to reconstruct gene structure using StringTie (ver. 1.3.5, download date: 17 August 2017, Sam Kovaka, Baltimore, MD, USA) with reference annotation and *-j 5 -c 5*. After merging GTF annotations, the final GTF file was compared with the reference annotation and the transformed gene id. StringTie was re-run to calculate the gene expression level with the final gene annotation. To compare the gene expression level under different environments, featurecount in Rsubread package (ver. 1.32.4, download date: 27 April 2019, Yang Liao, Parkville, Victoria 3052, Australia) was used to count RNA-Seq reads and the number was normalized using DESeq2 (ver. 1.22.2, download date: 9 March 2019, Michael I Love, Boston, MA, USA); the likelihood ratio test was used for differential expression analyses. Genes with Bonferroni-corrected *p* < 0.05 and | log2 (Fold Change) | > 1 were considered DEGs. The GO analysis of DEGs was performed by agriGO v2 with maize locus ID v3.30 as reference.

### 5.5. Identification of Alternative Splicing Events

For differential splicing analysis, Salmo (ver. 0.9.1) [55] was used for transcript quantification and SUPPA2 (ver. 2.3, download date: 7 February 2018, Juan L. Trincado, E08003, Barcelona, Spain) [56] was used for AS event identification with default settings. AS events with support reads > 5, PSI > 0.1, and PSI < 0.9 were retained and counted. To investigate the relationship between DNA methylation and AS, genes with different AS numbers were randomly sampled by fragments per kilobase million (FPKM), to ensure the exclusion of effects of gene expression. The software SUPPA2 also was used to analyze differential splicing under WS and splicing with *p* value < 0.05 was considered as a significantly differential AS event. The representative AS events in AC7643_DT and AC7729/TZSRW_DS were visualized using IGV browser (ver. 2.3.31, download date: 8 August 2017, James T Robinson, Cambridge, MA, USA).

### 5.6. siRNA Abundance and Their Fold Change

After removing adaptor sequences and aligning reads to microRNA (miRNA) precursor (miRbase ver. 22.1, download date: 7 June 2017, Ana Kozomara, Manchester M13 9PT, UK), small RNA reads were aligned to the maize Refv4 genome using bowtie (ver. 1.1.2, download date: 17 June 2015, Ben Langmead, College Park, MD, USA) with *-a -m 50 -best*. The siRNA reads associated with TE and genes were counted using featurecount in Rsubread package, and siRNA abundance was calculated as follows:

(1) $$siRNA\;TPM=\frac{ReadsCount}{\left(\frac{LibSize}{10^{6}}\right)}$$

The average siRNA abundance in the 2 kb flanking regions of genes or TEs was calculated in 100-bp windows with a 20-bp step. Correlation analysis was conducted using the cor.test function in R (ver. 3.5.1, download date: 24 December 2018, R Core Team, Vienna, Austria). For differential expression analysis, siRNA reads were normalized and compared with DESeq2 as the RNA-Seq analysis pipeline. Bonferroni-corrected *p* < 0.05 and | log2(Fold Change) | > 1 were set as the threshold for differentially expressed under WS.

## Figures and Tables

**Table 1:** The number and proportion of genes with altered DNA methylation.

	Hyper-Methylated DMRs			Hypo-Methylated DMRs		
	TE-related genes	Non-TE genes	*P* value (χ^2^ test)	TE-related genes	Non-TE genes	*P* value (χ^2^ test)
Genes	2963 (8.94%)	501 (3.97%)	5.44E72	1708 (5.15%)	353 (2.80%)	2.32E27
Exon	1563 (4.72%)	313 (2.48%)	5.74E27	923 (2.78%)	245 (1.94%)	3.78E07
Intron	1877 (5.66%)	291 (2.31%)	2.06E51	1202 (3.63%)	211 (1.67%)	4.64E27
Promoter 2k	3050 (9.20%)	986 (7.81%)	3.09E06	1478 (4.46%)	500 (3.96%)	2.08E02
Promoter 2k–5k	4469 (13.48%)	1363 (10.80%)	1.63E14	2349 (7.09%)	843 (6.68%)	1.32E01
Downstream 2k	2874 (8.67%)	696 (5.51%)	2.86E29	1636 (4.94%)	397 (3.15%)	1.22E16
Downstream 2k–5k	3682 (11.11%)	936 (7.42%)	1.25E31	2025 (6.11%)	589 (4.67%)	3.23E09

**Table 2:** The number and proportion of drought responsive genes in four maize inbred lines.

	AC7643_DT		AC7729/TZSRW_DS		RIL208_DT		RIL64_DS	
	Up-regulated	Down-regulated	Up-regulated	Down-regulated	Up-regulated	Down-regulated	Up-regulated	Down-regulated
Total genes	3588 (7.84%)	3975 (8.69%)	5180 (11.32%)	3920 (8.56%)	2817 (6.15%)	1791 (3.91%)	1927 (4.21%)	1930 (4.22%)
TE-related genes	2586 (7.80%)	2755 (8.31%)	3641 (10.98%)	2720 (8.21%)	2215 (6.68%)	1242 (3.75%)	1439 (4.34%)	1347 (4.06%)
*p* value	0.855	0.065	0.145	0.075	0.003	0.238	0.379	0.295
Non-TE genes	1002 (7.94%)	1220 (9.67%)	1539 (12.19%)	1200 (9.51%)	602 (4.77%)	549 (4.35%)	488 (3.87%)	583 (4.62%)
*p* value	0.726	0.001	0.007	0.001	0.000	0.029	0.091	0.051
*p* value (TE-related/Non-TE)	0.638	0.000	0.000	0.000	0.000	0.003	0.026	0.009

## Data Availability

The raw MeDIP, transcriptome and smRNA sequencing data of four lines were deposited in the the SRA database (accession number: SRP063383).

## Supplementary Materials

The following are available online at https://www.mdpi.com/article/10.3390/ijms22158285/s1.
