# Supplementary material for: Maize DNA Methylation in Response to Drought Stress Is Involved in Target Gene Expression and Alternative Splicing
Source: Int J Mol Sci. 2021 Jul 31;22(15):8285. doi: 10.3390/ijms22158285 (PMC8347047; doi:10.3390/ijms22158285)
Supplement: Supplementary file 1 [file ijms-22-08285-s001.zip › Supplemental Materials.pdf]

# Maize DNA Methylation in Response to Drought Stress Is Involved in Target Gene Expression And Alternative Splicing

## Supplementary materials

Table S1. Summary of MeDIP-seq in maize roots

Table S2. GO term enrichment of genes with DMR in promoter

Fig. S1. Genome-wide distribution pattern of Genes, TE, *copia*, *gypsy*, *helitron*, *TIR*, unknown transposon and DNA methylation peaks (from outer to inner ring). The number of genetic elements per Mb has plotted along with chromosomes.

Fig. S2. The proportion of random segments and DNA methylation peaks in the genomic regions (A) and various classes of TEs (B).

Fig. S3. Distribution pattern of GC content and DNA methylation peaks around the TSS (Transcription/TE Start Site) and TES (Transcription/TE End Site). (A) GC content of different genes. (B) GC content of genes and TE. (C) DNA methylation peaks distribution of genes and TE. (D) GC content of different classes of TEs.

Fig. S4. Highly conserved DNA methylation. Venn diagram of DNA methylation peaks under WW and WS (A), in drought-tolerant inbred lines and drought-sensitive (B), in the parental lines and RILs (C). Venn diagram of DNA methylation peaks of four materials under WW (D) and WS (E).

Fig. S5. DNA methylation can be inherited. (A) Bin map of RIL208_DT (top) and RIL64_DS (below). (B) Pie plot of consistency of bin map and SNPs from RNA-seq data. (C) The histogram of proportion of DNA methylation identical with corresponding parents or another parent.

Fig. S6. The number of intersecting sets of hyper- or hypo- methylated DMRs along with four inbred lines.

Fig. S7. The proportion of DMRs located in different classes of TEs.

Fig. S8. (A) Gene expression in eight samples. (B) DNA methylation distribution pattern of genes with different expression levels.

Fig. S9. (A) The DNA methylation reads coverage are plotted along exon junction. (B) The expression of genes with different alternative splicing events. (C) The number of MeDIP reads in four groups classified by the number of RNA-seq reads in exon of multiple-exon genes. (D) PSI of retained intron with and without DNA methylation

peaks overlapping.

Fig. S10. (A) The expression level of genes with and without TE overlapping. (B) The average DNA methylation peaks coverage are plotted along gene region as well as the upstream (-2 kb) and downstream (+2 kb) in Non-TE and TE-related genes with different expression levels. (C) The expression level of genes overlapped varying classes of TEs. (D) DNA methylation distribution pattern of genes overlapped different classes of TEs.

Fig. S11. (A) The heatmap of fold change (FC) of genes involved in DNA methylation/demethylation pathway under WS. (B) A higher proportion of DEGs in genes potentially regulated by DDM1 under water stress.
