# Supplementary figures and images for: Maize DNA Methylation in Response to Drought Stress Is Involved in Target Gene Expression and Alternative Splicing
Source: Int J Mol Sci. 2021 Jul 31;22(15):8285. doi: 10.3390/ijms22158285 (PMC8347047; doi:10.3390/ijms22158285)

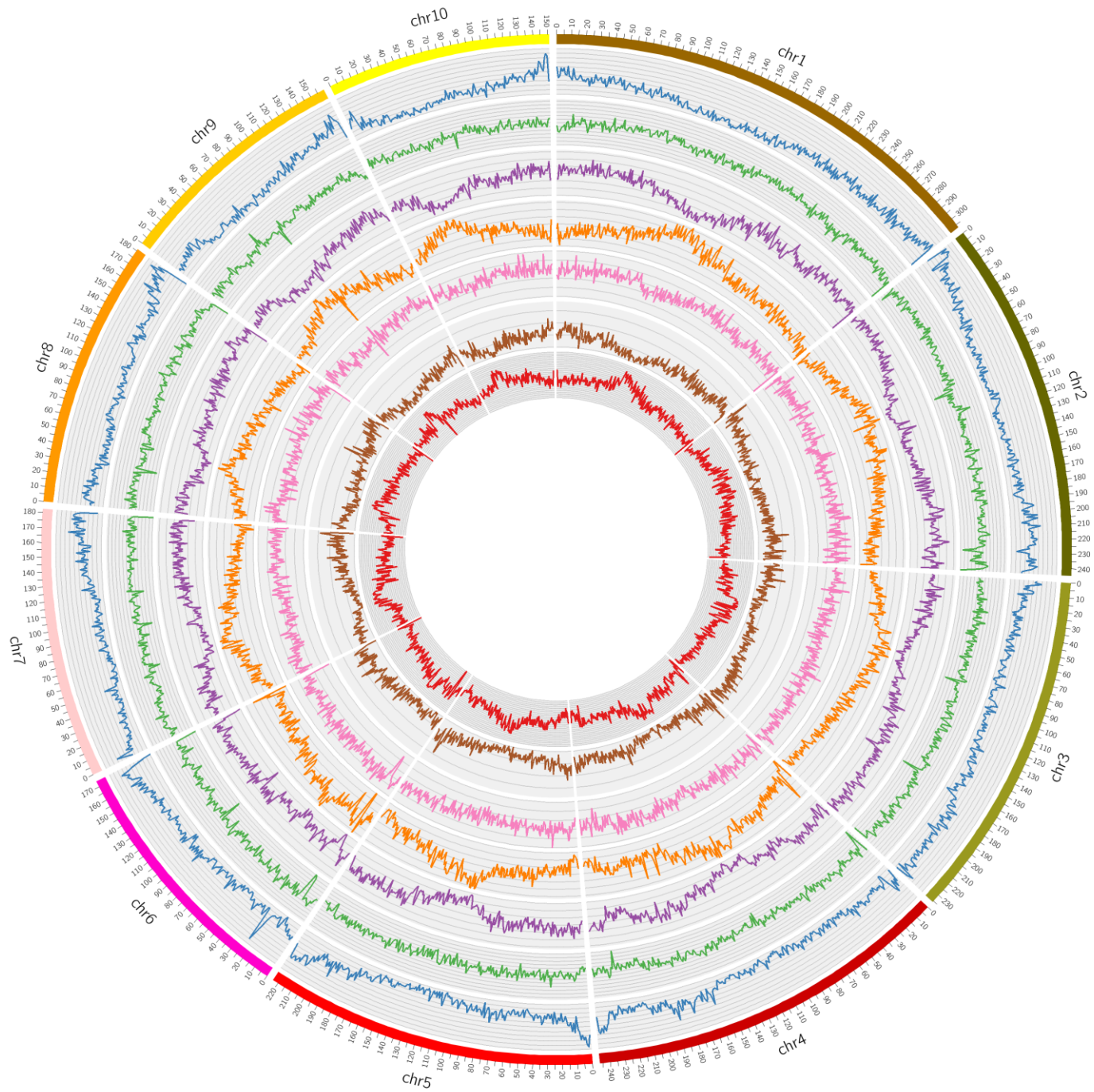

A

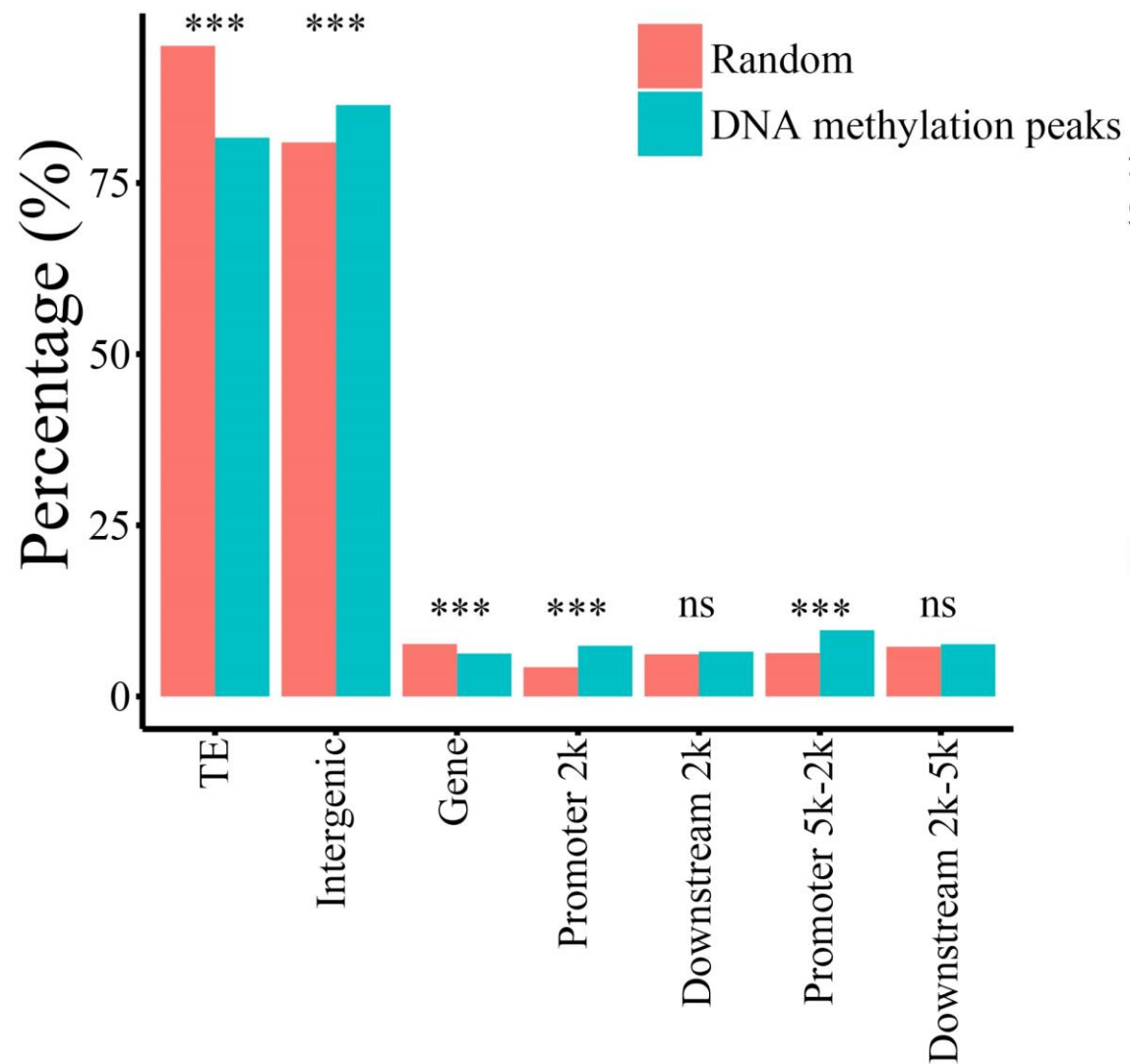

B

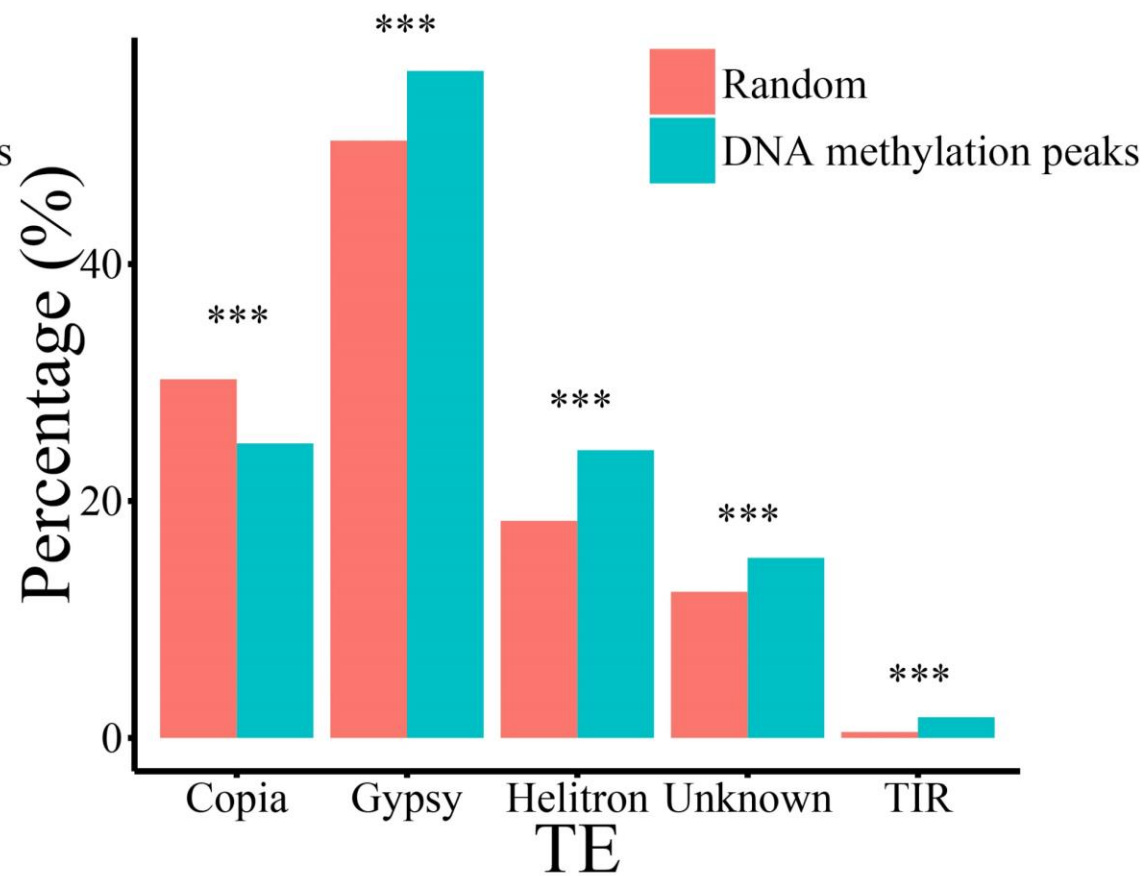

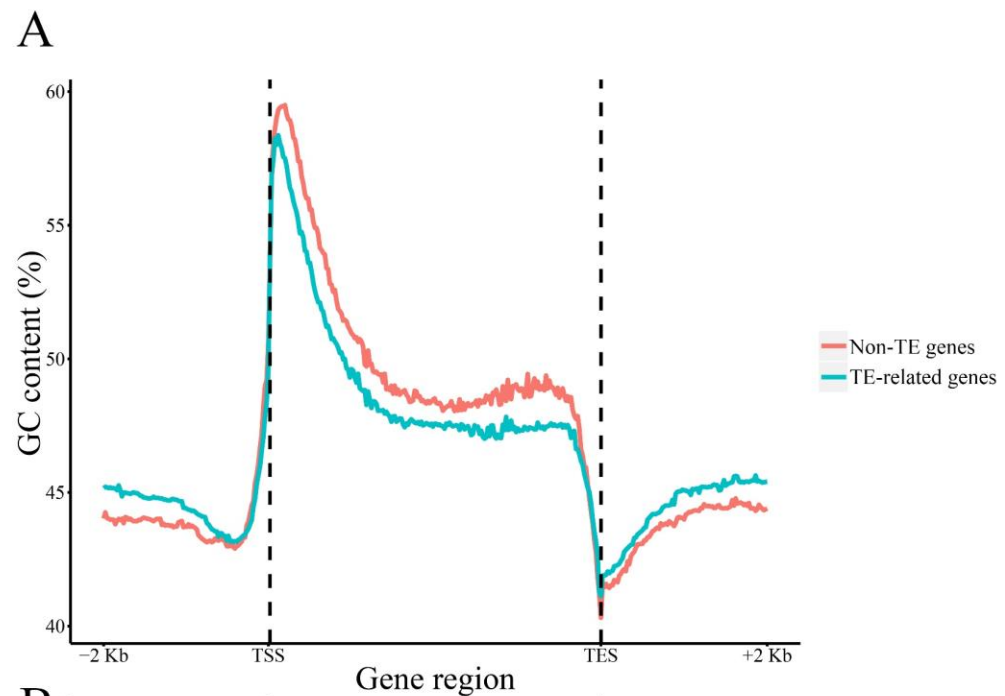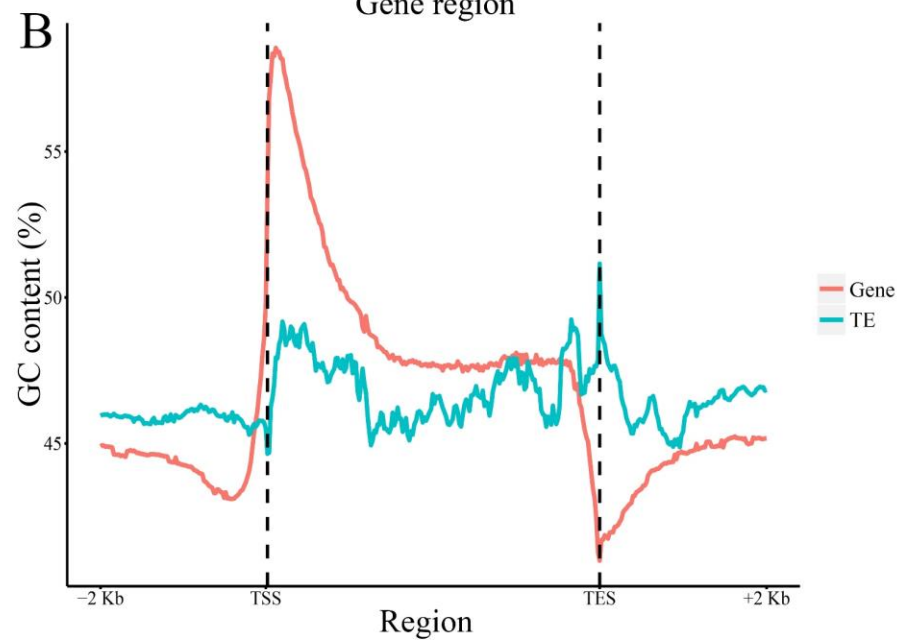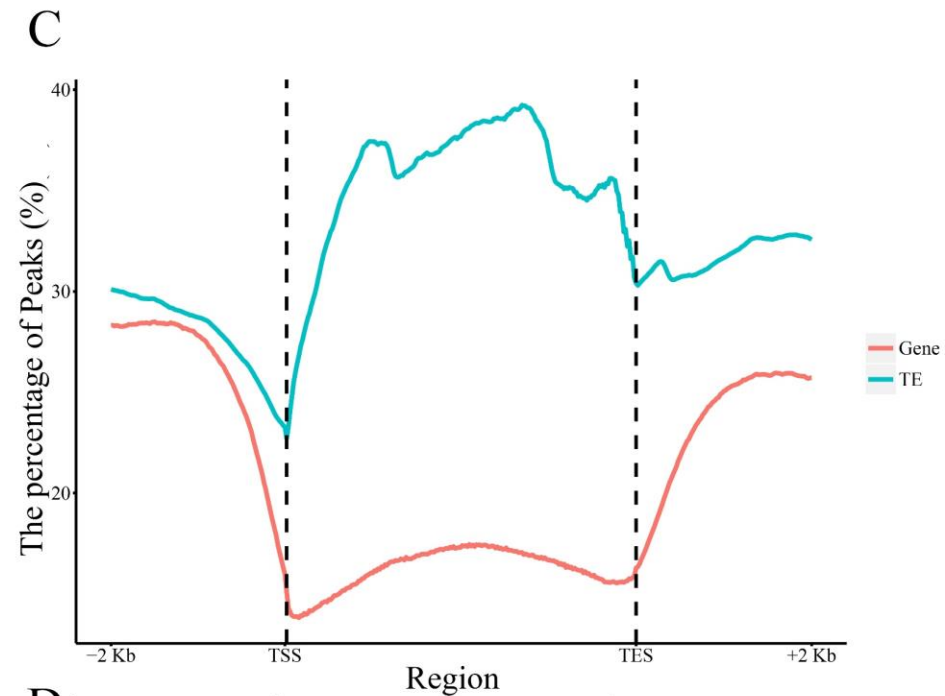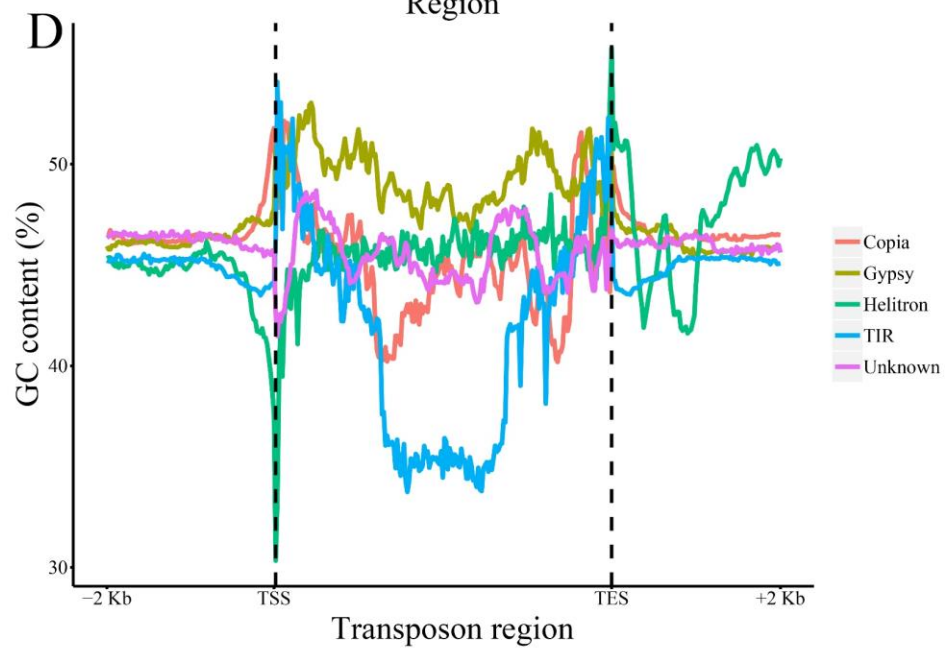

**A**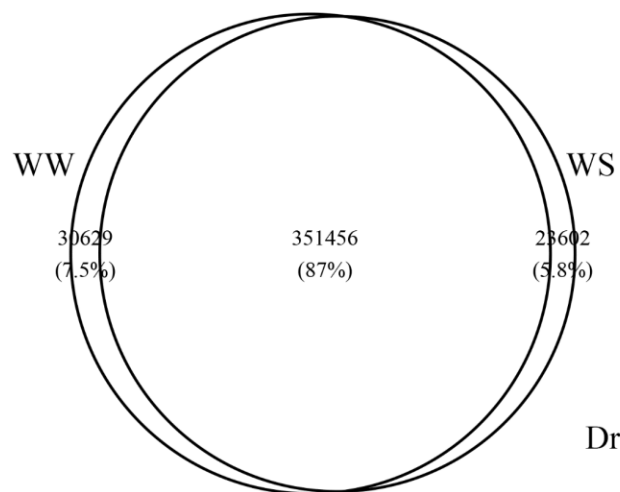**B**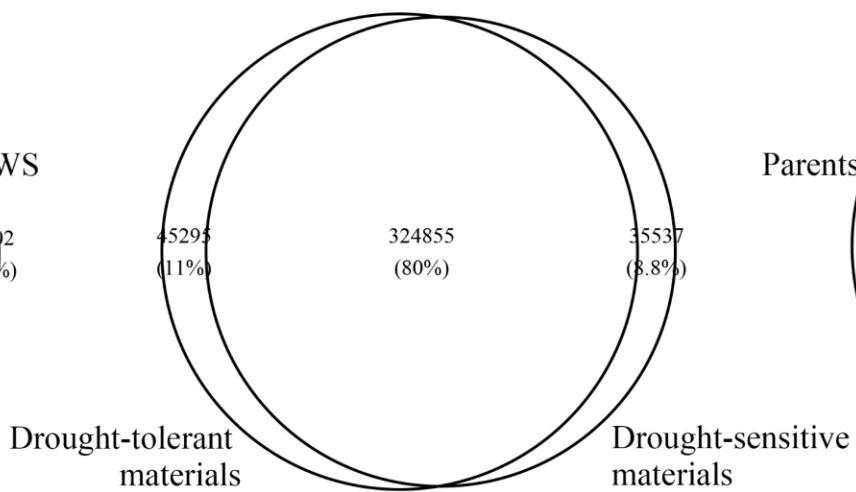**C**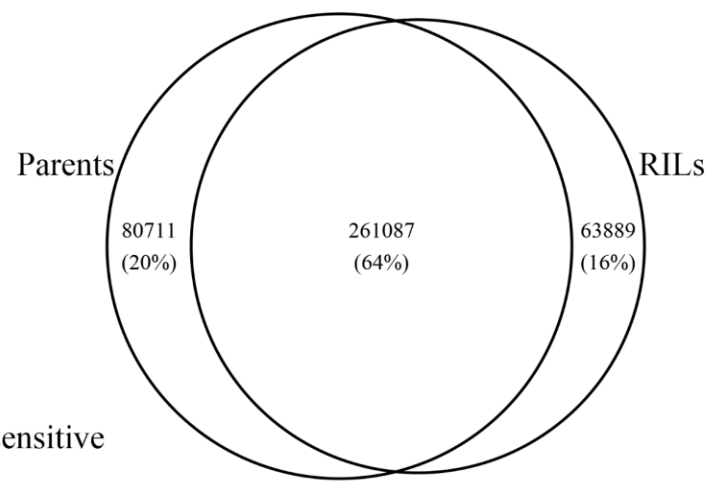**D**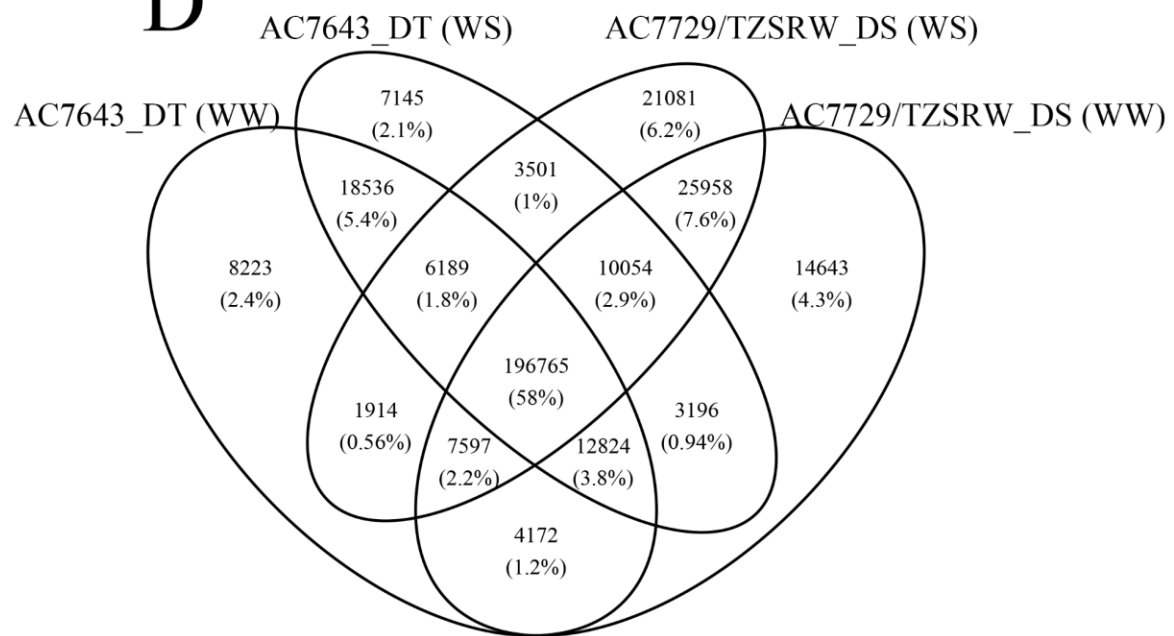**E**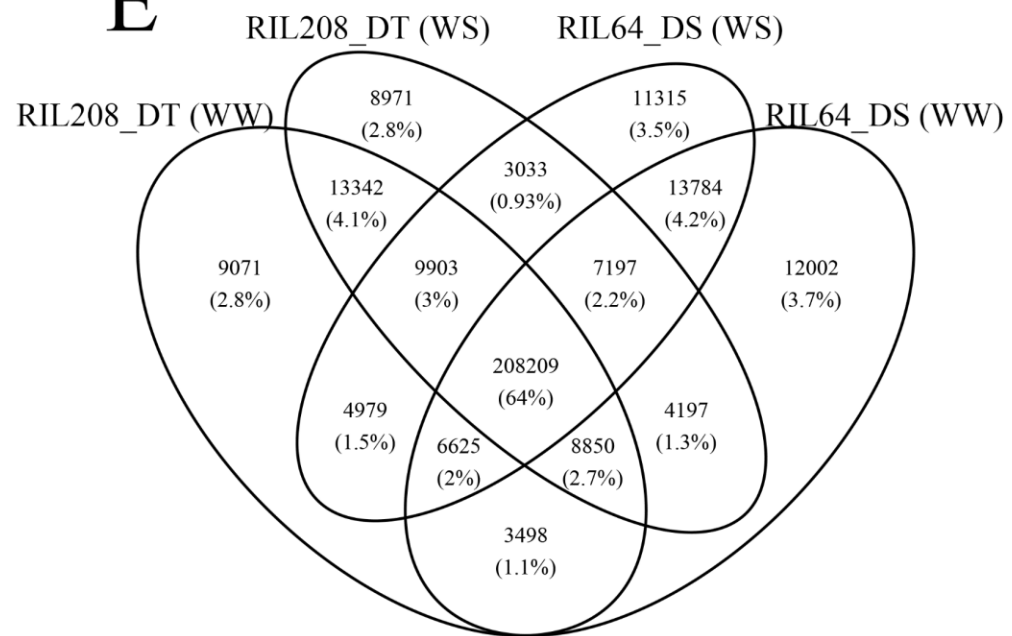

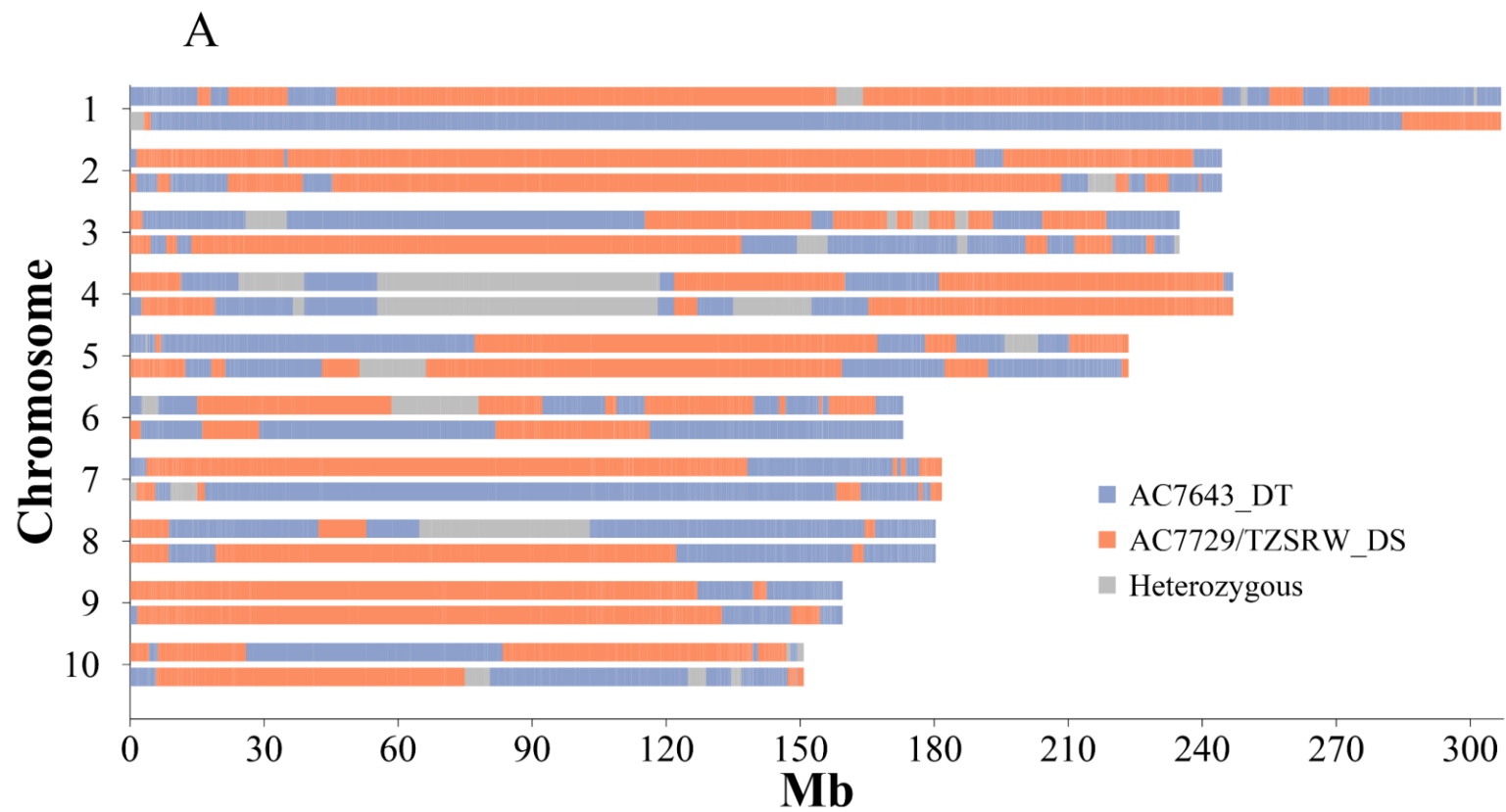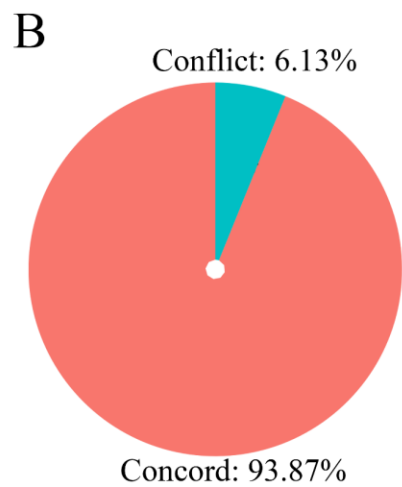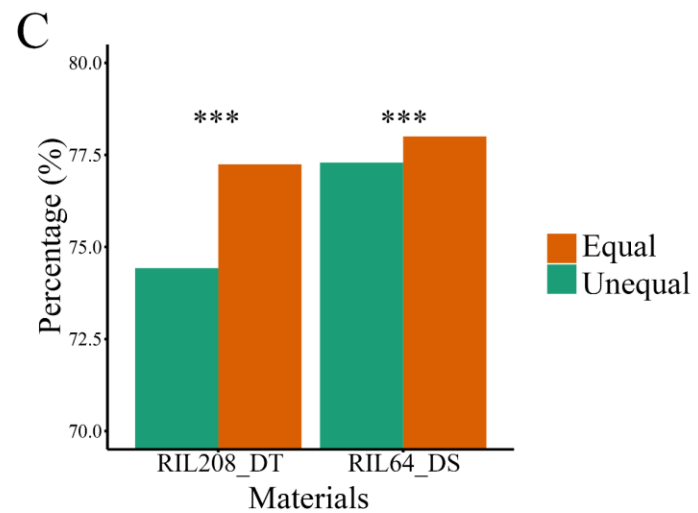

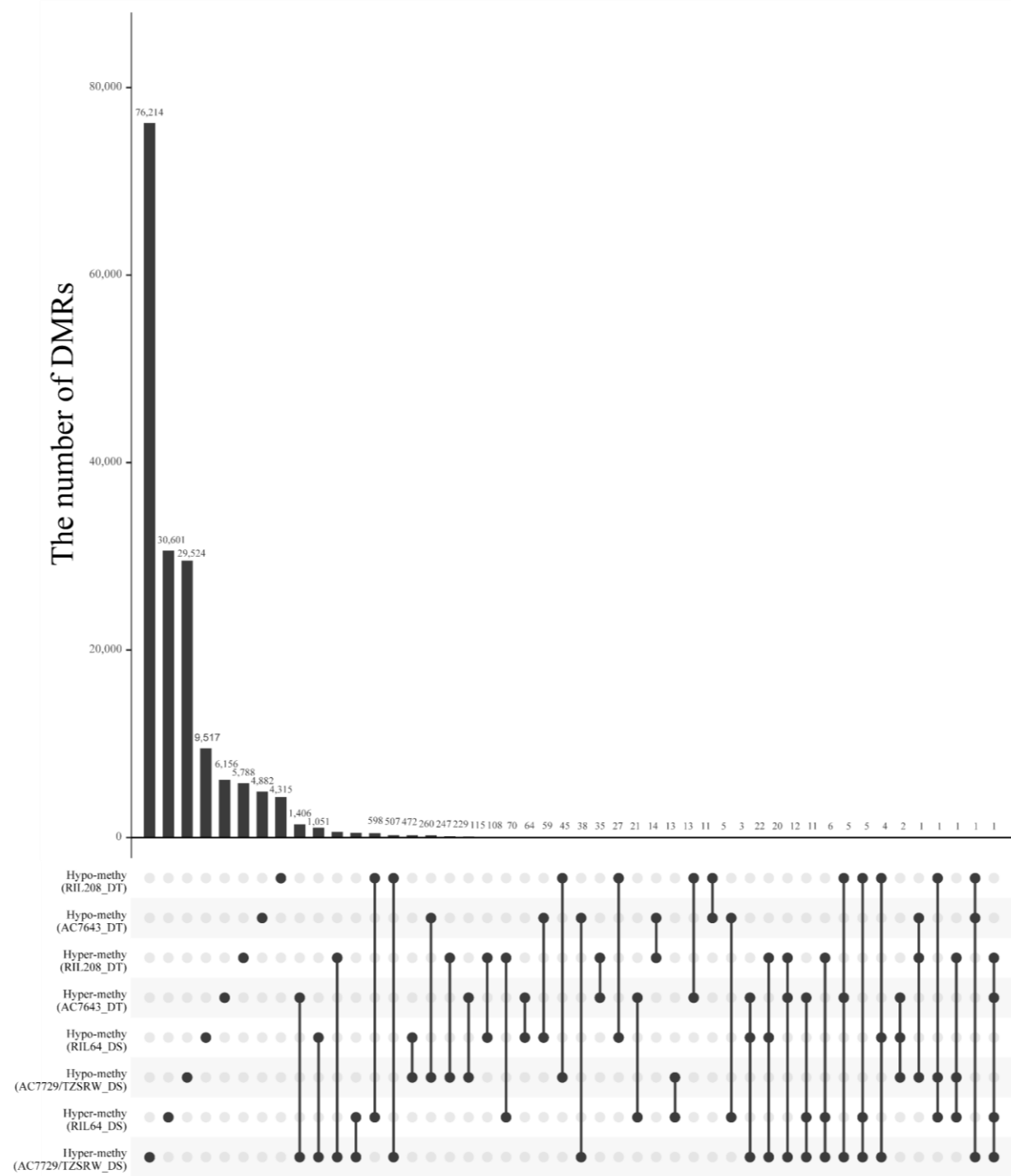

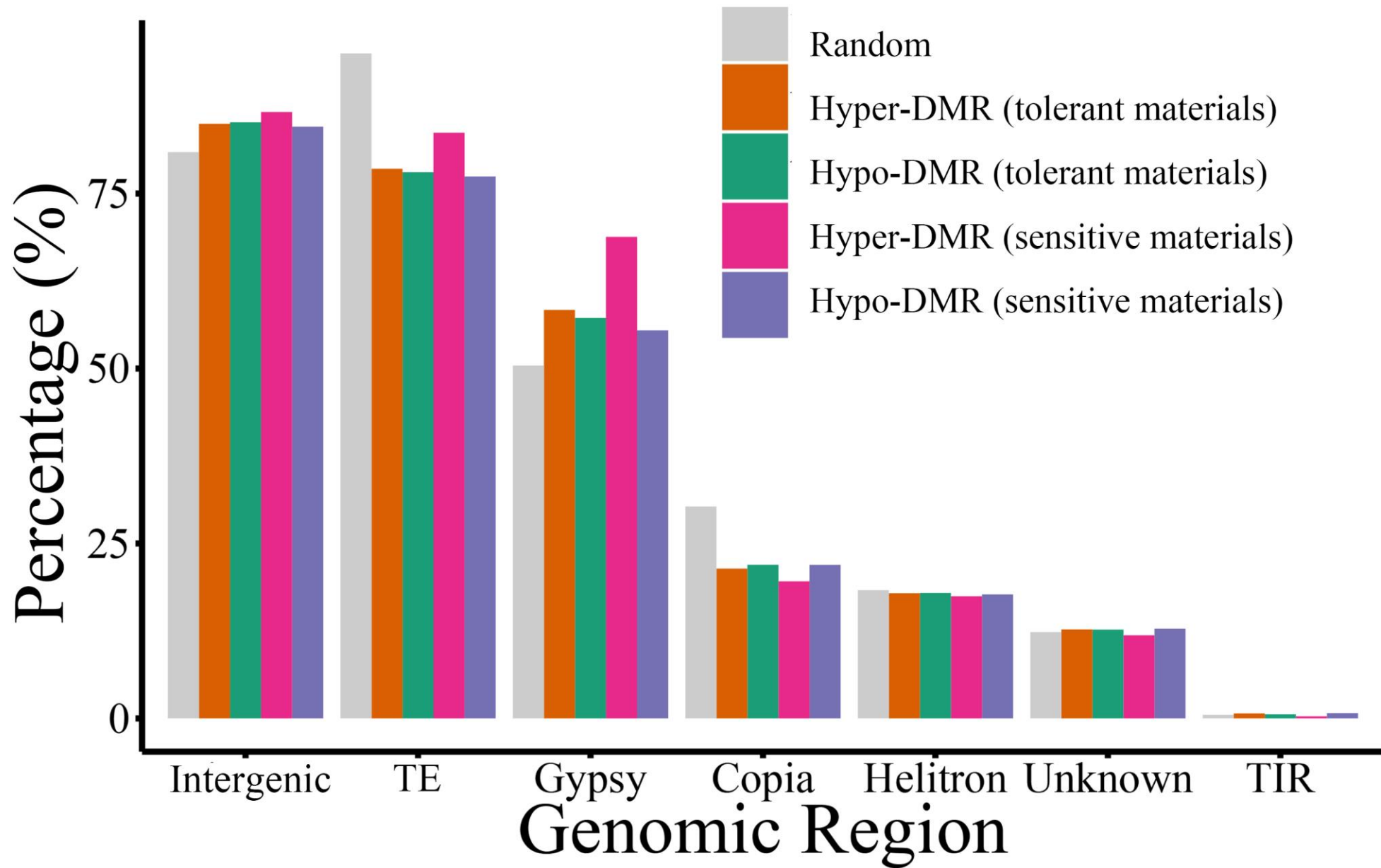

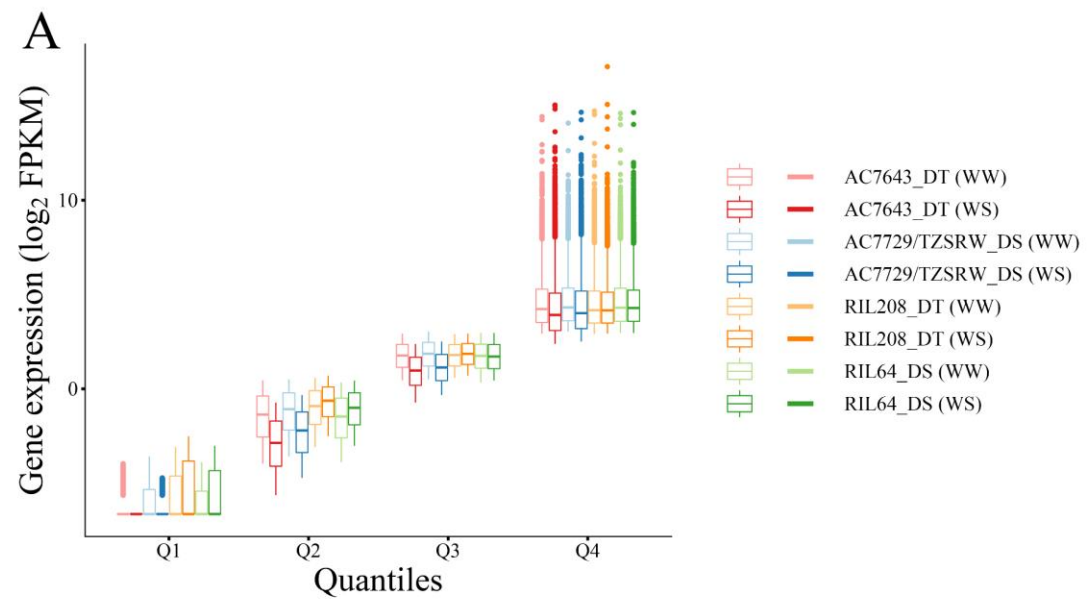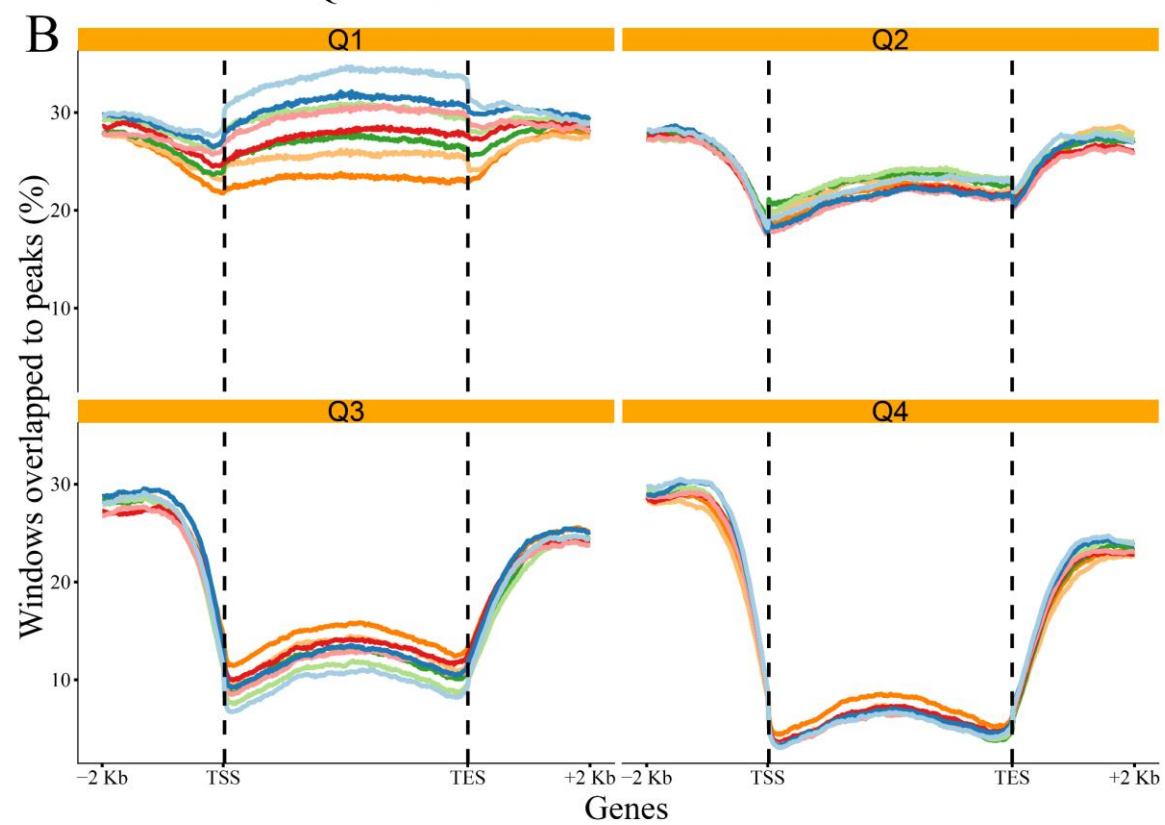

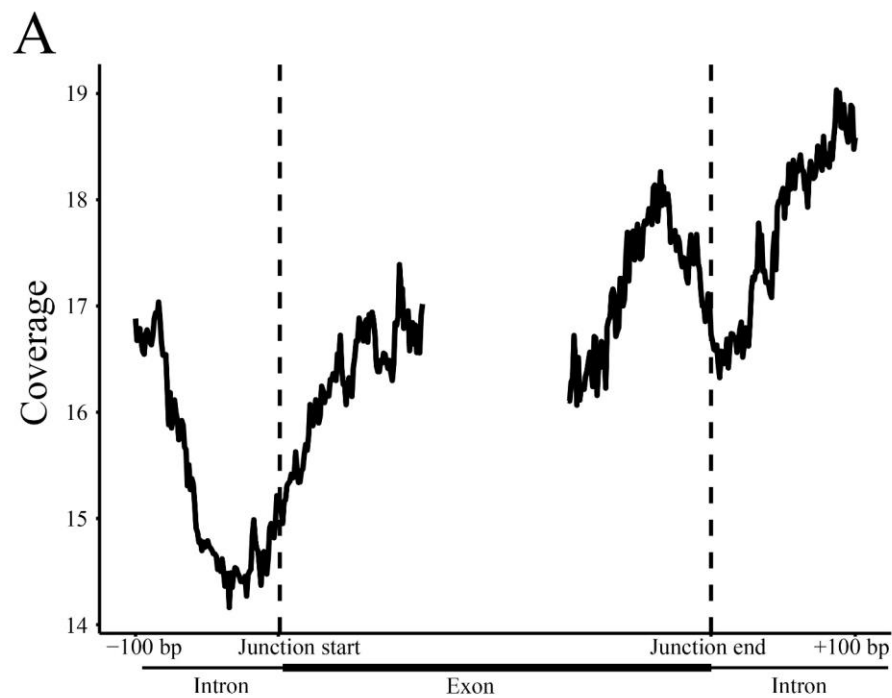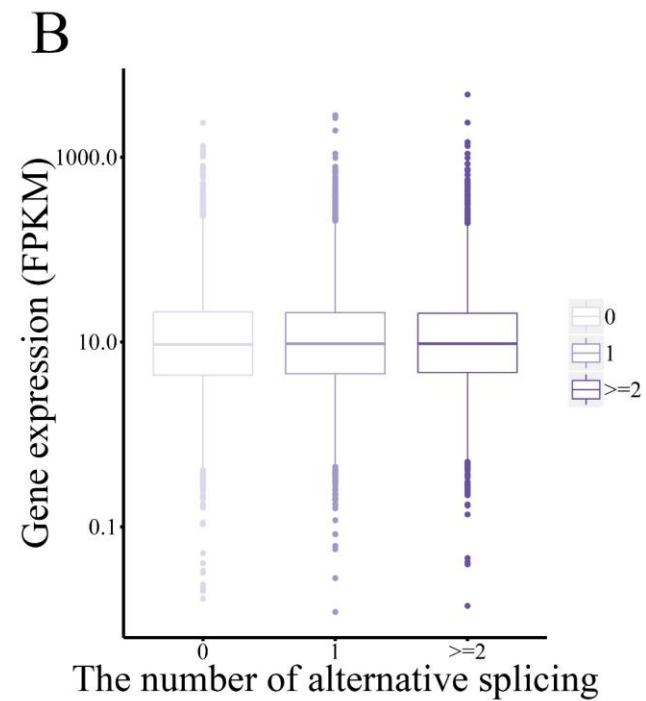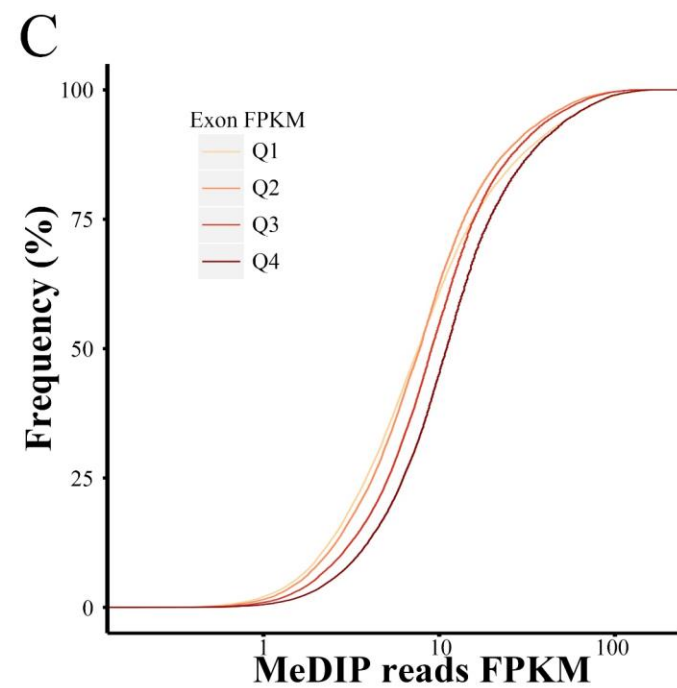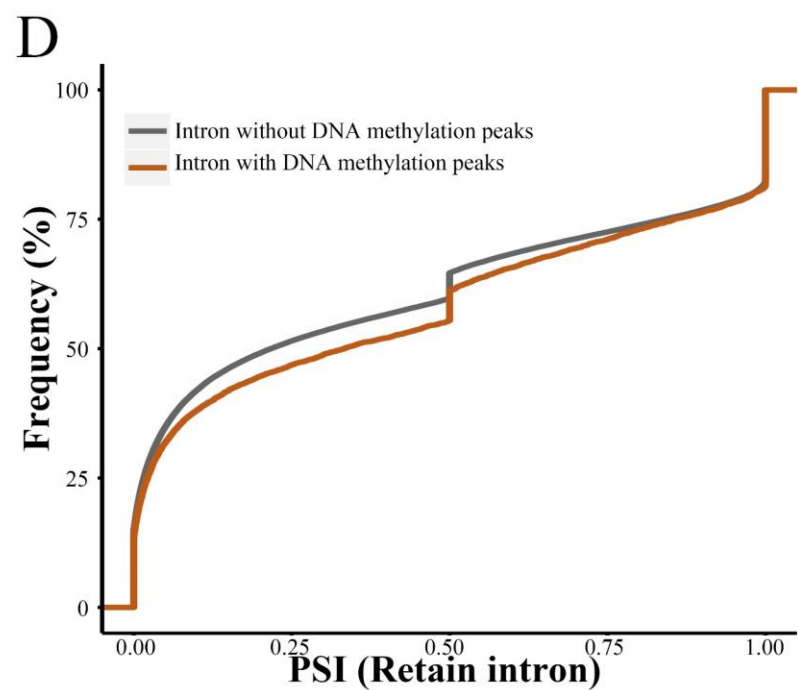

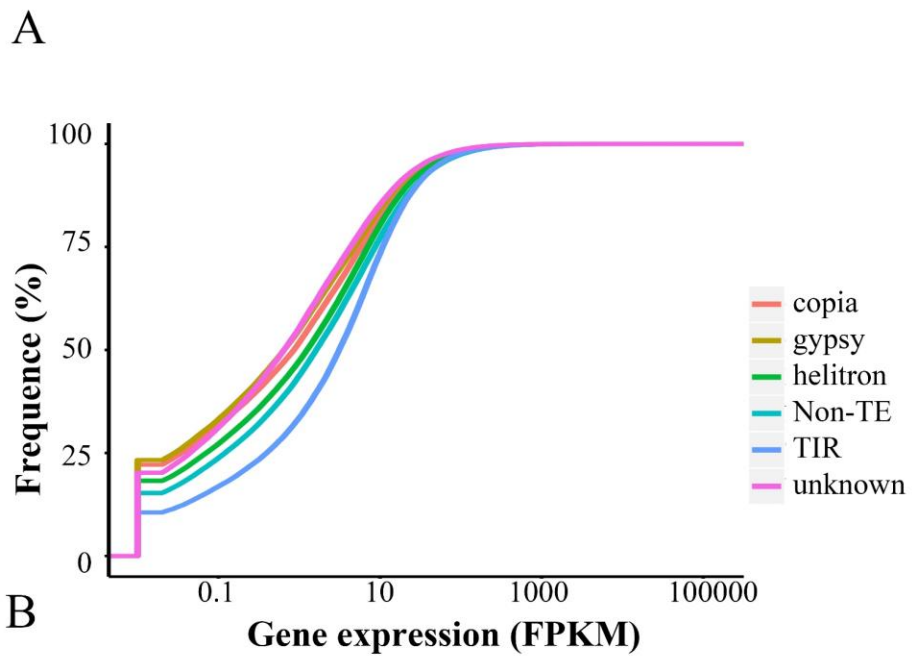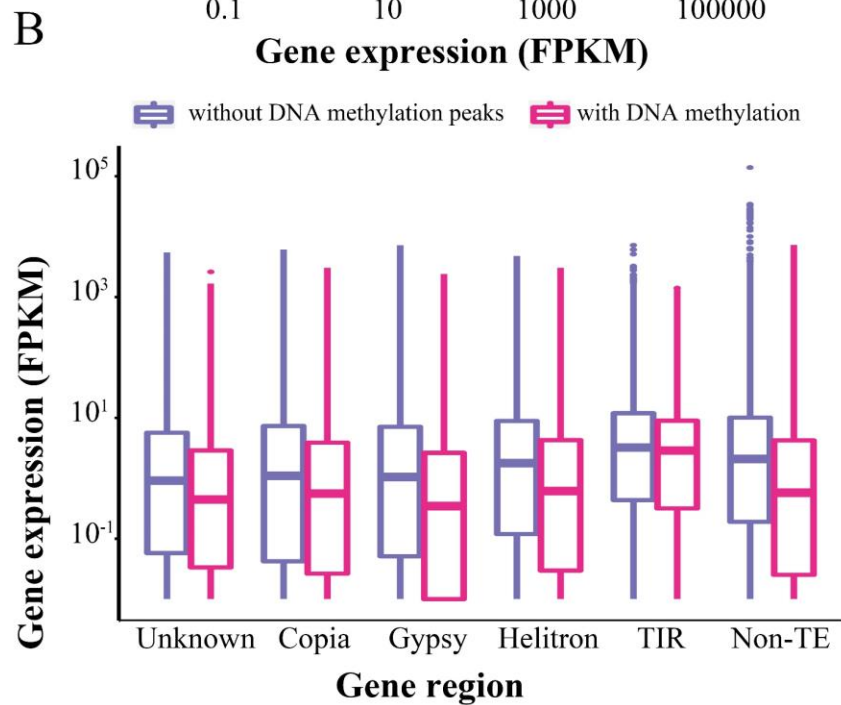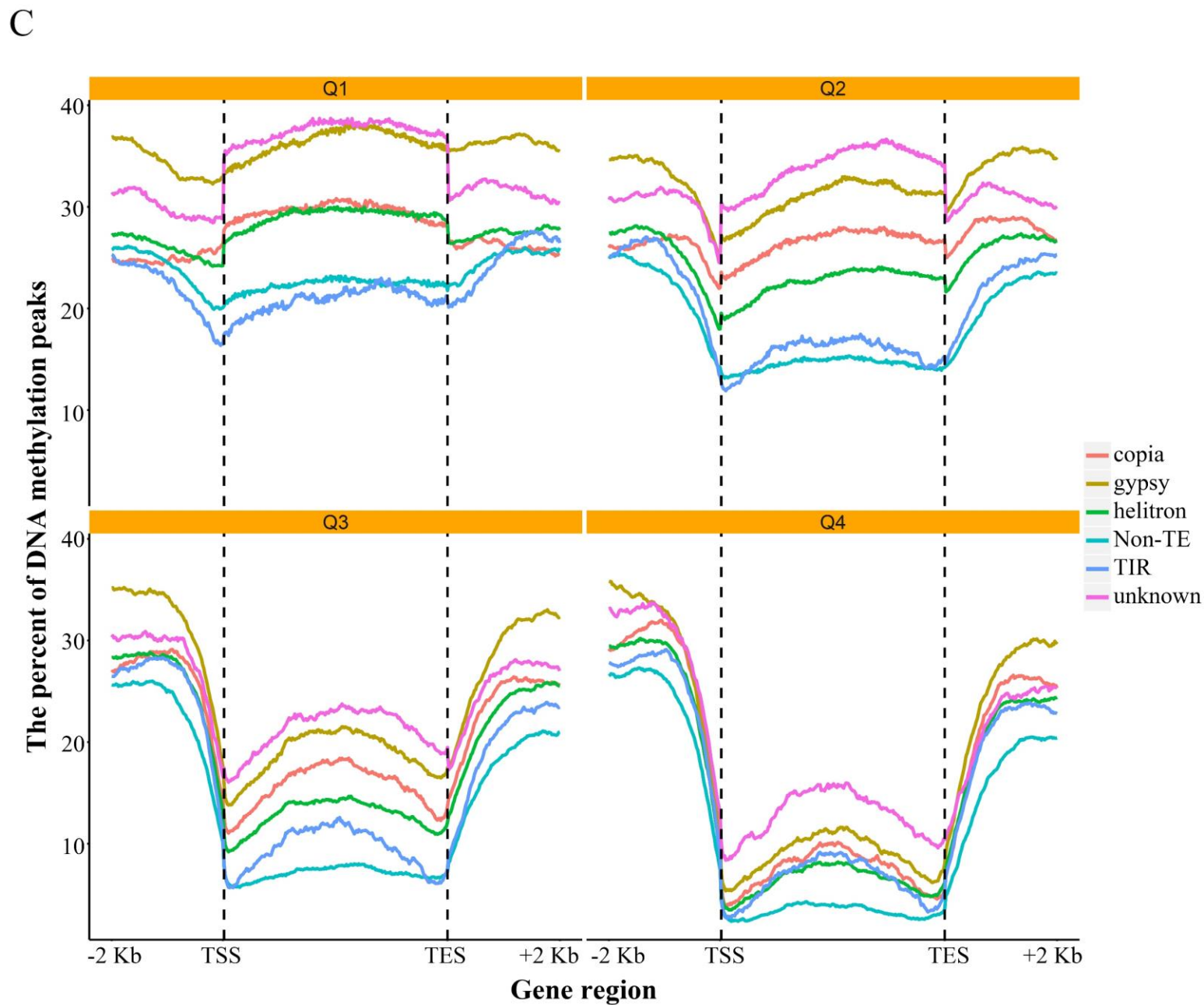

A

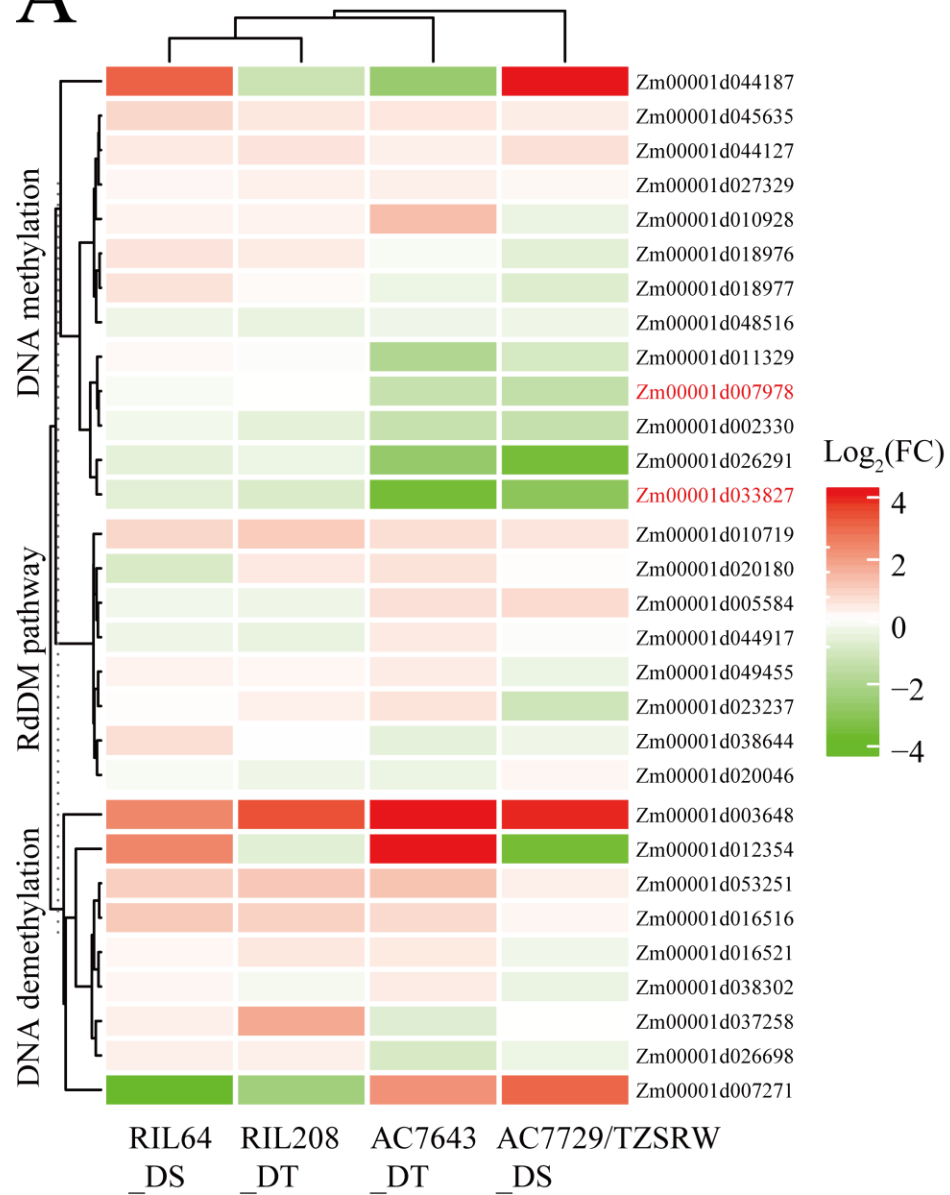

B

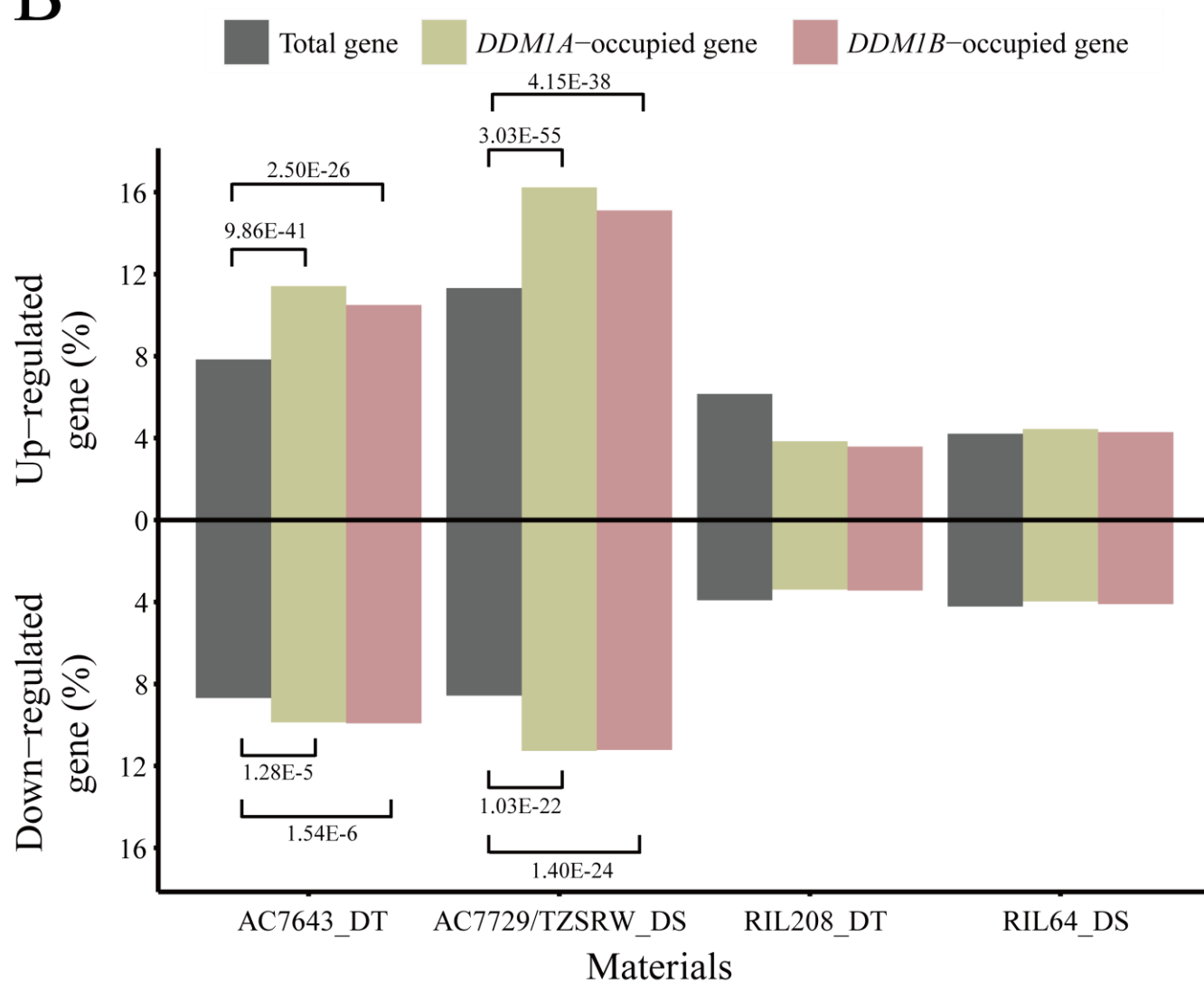

Supplement: Supplementary file 1 [file ijms-22-08285-s001.zip › Supplemental Figure.pdf]
